# Joint Analysis of Dependent Features within Compound Spectra Can Improve Detection of Differential Features

**DOI:** 10.3389/fbioe.2015.00129

**Published:** 2015-09-24

**Authors:** Diana Trutschel, Stephan Schmidt, Ivo Grosse, Steffen Neumann

**Affiliations:** ^1^Department of Stress and Developmental Biology, Leibniz Institute of Plant Biochemistry, Halle, Germany; ^2^Institute of Computer Science, Martin Luther University Halle-Wittenberg, Halle, Germany; ^3^German Centre for Integrative Biodiversity Research (iDiv) Halle-Jena-Leipzig, Leipzig, Germany

**Keywords:** metabolomics, statistics, hypothesis tests, multivariate analysis, mass spectrometry

## Abstract

Mass spectrometry is an important analytical technology in metabolomics. After the initial feature detection and alignment steps, the raw data processing results in a high-dimensional data matrix of mass spectral features, which is then subjected to further statistical analysis. Univariate tests like Student’s *t-*test and Analysis of Variances (ANOVA) are hypothesis tests, which aim to detect differences between two or more sample classes, e.g., wildtype-mutant or between different doses of treatments. In both cases, one of the underlying assumptions is the independence between metabolic features. However, in mass spectrometry, a single metabolite usually gives rise to several mass spectral features, which are observed together and show a common behavior. This paper suggests to group the related features of metabolites with CAMERA into compound spectra, and then to use a multivariate statistical method to test whether a compound spectrum (and thus the actual metabolite) is differential between two sample classes. The multivariate method is first demonstrated with an analysis between wild-type and an over-expression line of the model plant *Arabidopsis thaliana*. For a quantitative evaluation data sets with a simulated known effect between two sample classes were analyzed. The spectra-wise analysis showed better detection results for all simulated effects.

## 1 Introduction

Mass spectrometry is an important analytical technology in metabolomics. XCMS (Smith et al., 2006) is one of the available tools for processing mass spectrometry data. After the initial feature detection and alignment steps, the raw data processing results in a high-dimensional data matrix of mass spectral features as shown in Table 1, which is then subjected to further (statistical) analysis.

**Table 1:** **A peak list of features of a two sample class MS experiment with feature group annotation mz is the mass-to-charge ratio, RT is the retention time in seconds**.

mz/RT	MU 1	MU 2	…	MU 6	MU 7	WT 1	WT 2	…	WT 6	WT 7	p.uni	group.anno	p.multi
590.5/967	14.42	14.61	…	14.29	14.2	13.85	13.96	…	13.95	14.12	0.02	40	$\begin{array}{l} \;\\ \;\\ \; \end{array}\}0.02$
609.5/968	18.31	18.72	…	18.32	18.45	18.12	18.7	…	18.44	18.48	0.88	40	
628.5/968	17.21	17.52	…	17.17	17.21	16.95	17.49	…	17.18	17.34	0.89	40	
…	…	…	…	…	…	…	…	…	…	…	…	…	
413.3/1106	14.92	13.23	…	14.72	14.57	14.52	14.92	…	14.52	14.27	0.65	82	$\begin{array}{l} \;\\ \;\\ \;\\ \;\\ \; \end{array}\}0.30$
538.5/1103	12.32	11.76	…	11.93	11.8	11.7	11.7	…	12.15	12.91	0.23	82	
591.5/1101	15.51	15.2	…	15.36	15.06	15.72	15.78	…	15.07	15.74	0.02	82	
592.5/1102	15.15	14.78	…	14.78	14.42	14.67	15.03	…	14.76	15.33	0.34	82	
797.5/1104	18.28	17.96	…	17.72	17.58	17.83	18.42	…	17.2	17.91	0.15	82	

*Additionally, listed uni- and multivariate *p*-values results from univariate and multivariate tests*.

A typical question in metabolomics is biomarker discovery, where e.g., univariate hypothesis tests like Student’s *t*-test (Student, 1908) and Analysis of Variances (ANOVA) can be used to detect differences between two or more sample classes, e.g., wildtype versus mutant or disease versus control. An example implementation is the diffreport() function in XCMS. Furthermore, some statistical methods can deal with more complex experimental designs with dependencies between samples (Davis, 2002; Sampson et al., 2013; Trutschel et al., 2015). But in all cases, one of the underlying assumptions is the independence between individual metabolic *features*.

However, in mass spectrometry, a single metabolite usually gives rise to several mass spectral features, e.g., isotopes, adducts, or fragments (Brown et al., 2009), which observed together and show a common behavior across samples. Another issue is that the redundant features aggravate the problem of multiple testing, and cause more type I errors (Broadhurst and Kell, 2006; Hendriks et al., 2011).

A first step to treat related features together is to group those, which originate from the same metabolite into compound spectra. Several methods for such a grouping have been developed in the last years (Ipsen et al., 2010; Alonso et al., 2011; Brown et al., 2011; Scheltema et al., 2011; Varghese et al., 2012; Kenar et al., 2014). In this paper, the grouping algorithm in the Bioconductor package CAMERA (Kuhl et al., 2012) is used, which is comprised of several steps, including compound spectra creation based on retention time, calculation of known mass differences for isotope pattern and adduct detection and a peak shape correlation analysis. This grouping then results in *compound spectra*, which contain one or more related features, which originate from the same metabolite.

A typical approach for the statistical analysis in GC/MS is to select a single *quantification ion* for each compound (Luedemann et al., 2008) for univariate tests, ignoring intensity information for the remaining mass features in a compound spectrum. On the other hand, multivariate methods like MANOVA are global approaches and analyze all features together and can take correlations into account. This has already been used in metabolomics (Steuer et al., 2007; Saccenti et al., 2014). With MANOVA, the simultaneous analysis of variables results in a better Type I error correction because of the multidimensional confidence region. In more detail, the differences in the mathematical theory between univariate and the multivariaten comparison for more than two groups (ANOVA versus MANOVA) are described in (Legendre and Anderson, 1999). The multivariate approach benefits from small signals, which contribute to the class differences, but would not be detected univariate because the effect is too small compared to the variance. However, the interpretation, which metabolites have changed, remains challenging.

Often, in metabolomics, the number of samples is much smaller than the number of features to be analyzed. Therefore, correlation and covariance structure is difficult to estimate, and requires an initial variable selection step. Often, the complex models used by global multivariate analysis are prone to the problem of over-fitting with poor prediction and generalization.

In this paper, we compare the detection of differential features on the individual- and metabolites on the compound spectra level. We also introduce a multivariate analysis on the level of compound spectra instead of a global multivariate approach to determine differential metabolites, combining the benefits of uni- and multivariate analysis for biomarker detection. An advanced version of the XCMS diffreport() function is provided for users. This paper is structured as follows: in the next section, the metabolomics data used in this paper is briefly described, followed by the conceptual details of the statistical method. The method is applied to data from wild-type and over-expression plants. Finally, the performance of the proposed methods is compared to the univariate approach on a data set of known (simulated) effects. The implementation is provided as an R vignette in the Supplementary Material under the GPL license.

## 2 Materials and Methods

For the experiments, two metabolomics data sets from *Arabidopsis thaliana* (*A. th*.) were used. The first is a subset of the study available as MTBLS74, where 26 independent plant profiles and a simulated effect were used. The method is then demonstrated on a dataset of *A. th*. wildtype and a mutant line, available as MTBLS169.

### 2.1 Metabolite profiling of *Arabidopsis thaliana*

#### 2.1.1 Plant Growth and Sample Preparation

The model plant *Arabidopsis thaliana* Col-0 was used as plant material. For the genotype comparison Col-0 and the 90.32 mutant were used, a transposon-based activation tagged *A. th*. line from the TAMARA population (Schneider et al., 2005). This particular mutant has an over-expression of the AT5G55880 – AT5G55890 genetic region with unknown function. Plants were grown on soil in a growth chamber under controlled conditions as biological replicates. The frozen leaf material of each plant was ground and weighed using a cryogenics robot^1^ with a weighing error ≤5%, and extracted with methanol. Full details are available in Supplementary Material I, Section 1 and the protocol sections of the MetaboLights studies.

#### 2.1.2 Mass Spectrometry Analysis and Data Processing

Metabolite intensities were recorded according to (Böttcher et al., 2009). In brief, the chromatographic separation was performed on a Waters Acquity UPLC system coupled to a Bruker micrOTOF-Q mass spectrometer. Mass spectra were recorded in positive ion centroid mode with a scan rate of 3 Hz and a mass range of 100–1000 m/z. Full details are available in Supplementary Material I, Section 1 and the protocol sections of the MetaboLights studies. This experimental setup is able to routinely detect semi-polar plant metabolites from major biosynthetic classes including glucosinolates, indolic compounds, phenylpropanoids, benzenoids, flavonoids, terpenes, and fatty acid derivatives (Böttcher et al., 2011). In this paper, no metabolite identification was performed, resulting in the lowest metabolomics standards initiative (MSI) identification level (Sumner et al., 2007) MSI level four (i.e., the features are only characterized by their mass and retention time).

The measured MS data were converted to mzData with the Bruker CompassXport software. The mzData are preprocessed with the centWave feature detection algorithm (Smith et al., 2006; Tautenhahn et al., 2008) to condense the raw data to feature lists, and then aligned across samples to produce a matrix of *N* mass features observed in *M* samples. The xcms processing parameters are detailed in Supplementary Material I, Section 1, in particular, with minfrac = 1 no NA values were present in the *M* × *N* matrix to avoid any influence of a data imputation step in this evaluation. An underlying assumption of the original Student’s *t*-test (and also ANOVA) is that the mean intensities are normally distributed. To transform the data toward more normally distributed values, all intensities were logarithmized. The related features (rows in the matrix) are grouped into compound spectra with the package CAMERA. For the remaining analyses, this CAMERA grouping is assumed to be correct. Furthermore, there is no dependency on a CAMERA based grouping, and the proposed statistical treatment can be applied to groupings from equivalent tools as well.

The raw data files, the preprocessed peak matrix, and the protocol descriptions have been submitted to the MetaboLights repository (Haug et al., 2013), and are available under the accession number MTBLS74^2^. Analogously, the second data set is available as MTBLS169^3^. All statistical calculations were performed in (R Development Core Team, 2014). The complete processing scripts are provided in the Supplementary Material I, Section 1.

### 2.2 Detection of differential features and metabolites

The analysis for differential metabolites requires to detect intensity differences between sample classes. Here, in comparison to univariate methods to analyze features, we propose several multivariate methods to analyze compound spectra representing metabolites. First, we introduce with a graphical illustration of the different decisions from univariate and multivariate tests, then we explain the several tests. All formulas of the test are shown in detail in the Supplementary Material I, Section 3.

#### 2.2.1 Univarate Tests

The univariate Student’s *t*-test (Student, 1908) assumes normal distributed observations of independent features. The difference of the intensity mean between the two classes is estimated for each feature. While Student’s *t*-test assumes equal variances of the two classes, the Welch’s *t*-test (Welch, 1947) is a variant that allows different variances between the classes (Table S1 in Supplementary Material I, Section 3).

The confidence interval (CI) determines the accuracy of this estimation, and the CI size depends on the number of observations and the standard error (SE) of the estimated difference between means. The null hypotheses, *H*_o_, is that no difference in means exists, the alternative *H*_1_corresponds to a difference in means. If the CI includes the origin (zero), then the difference is considered not significant and *H*_o_ can be accepted.

**Figure 1:**
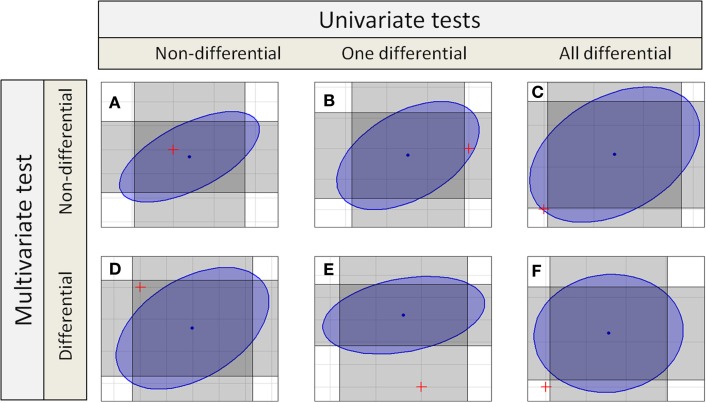
**Different decisions from univariate and multivariate test to detect differential features or compound spectra**. Each gray rectangles marks the confidence interval of one test dimension, so the intersection of two rectangles marks the combined confidence region. The blue ellipse is the confidence region for a multivariate test. There are six different possibilities (six different colored spaces) for the position of the origin corresponding to the null hypotheses marked by a red “+.”

If independent univariate tests for two features in a compound spectrum are combined, the confidence interval becomes a rectangular confidence region as shown in Figure 1, or in general for groups with *p* features a *p*-dimensional hypercube. Even if multiple testing correction is done, the confidence region holds a hypercube.

#### 2.2.2 Multivariate Tests

The multivariate extension of Student’s *t*-distribution was introduced by (Hotellings, 1931). The two-sample test of unequal means with unknown and equal variances becomes in multiple dimensions the Hotelling’s *T*^2^ (c.f. Table S1 in Supplementary Material I, Section 3). For unequal covariance matrices, the extension of the Welch t-test, is the James test (Table S1 in Supplementary Material I, Section 3), introduced in James (1954).

These tests compare the difference of *p*-dimensional mean intensity vectors in relation to their *p* × *p* covariance matrices. Observations of features in a compound spectrum are then assumed to be multidimensional normal distributed. For this multivariate analysis, the confidence region has an ellipsoid shape.

Using the multivariate tests, this statistic requires at least (*p* + 1/2) replicates, where *p* is the number of features per metabolite group, to estimate the unknown entries of each covariance matrix. For typical experiments, *p* easily exceeds 20 for some metabolite groups, but data sets with so many replicates are rare.

In the following, we additionally propose a variant of the multivariate methods, where only the diagonal entries of the covariance matrix are estimated, with the rest fixed to zero. This simplification ignores the correlation between features, but makes the covariance estimation more robust in the case where a compound spectrum consists of more features than samples are available to modify the idea of spectra-wise analysis on small data sets. The main axes of the ellipsoid confidence region are then parallel to the coordinate axes. The details and comparison of all tests are given in Table S1 in Supplementary Material I, Section 3.

#### 2.2.3 Comparison of Results from Univariate and Multivariate Tests

Depending on the univariate or different multivariate test statistics different sets of metabolic compound spectra are detected as differential. The *H*_o_ hypothesis is accepted if the assumed difference in means of zero between sample classes falls within the confidence interval or region. Several regions are shown in Figure 1.

The table also shows the different possible results for compound spectra with two features. In the simplest cases, both approaches yield the same result: in case of Figure 1A, no feature is differential using the univariate tests, and the compound spectrum as a whole is also not detected as differential by the multivariate test. Similarly, in Figure 1F, all features of the compound spectrum are differential in the univariate tests and the compound spectrum is assigned as differential by the multivariate test. But there are also cases, where the results completely differ: In Figure 1C, all features of the compound spectrum are differential in the univariate case, but the compound spectrum is not assigned as differential by the multivariate test, while in Figure 1D, none of the individual features is differential but the whole compound spectrum is detected as differential by the multivariate test. Finally, in Figures 1B,E, the two univariate tests for the individual features decide differently, and only one agrees with the multivariate test on the compound spectrum.

### 2.3 Evaluation data and performance measures

The distinction between differential and non-differential can be described as a classification problem and then the typical performance measures can also be used. For the evaluation, a ground truth data set is required, where for each feature, it is known whether it is differential or not. Then, the evaluation (Algorithm 1 in the Supplementary Material I) can assess the quality of biomarker discovery with the different statistical tests by calculating the confusion matrix and the derived measures specificity and sensitivity.

The ground truth used here is a real world data set with simulated (and hence known) effect between two classes. The data set of 26 *A. th*. Col-0 wildtype plants was randomly split into two sample classes, designated as “wildtype” and “mutant,” with 13 samples each.

To simulate differential features, for each compound spectrum an effect was sampled from a multivariate normal distribution with a given mean (determined by the desired effect, e.g., 0.5) and the covariance matrix that was estimated from the actual data in the 13 observations in the original “mutant class.” These effects were added to the features in the “mutant class.” This simulation ensures that effects are sampled for each separate compound spectrum (i.e., metabolite), rather than adding an effect to each feature individually. Thus, all compound spectra (and all its features) should be differential, and are the positive set of the ground truth. For the negative set of the ground truth, an “effect” of size zero was used.

For the simulation of the “mutant” class, only a subset of the available compound spectra can be used, since the sampling of an effect requires to estimate the covariance matrix of the compound spectra from 13 samples, which in turn is only possible for those compound spectra with a maximum of 12 features. For larger groups, it is impossible to parametrize the normal distribution used to simulate the fixed effect. Like wise, singletons (i.e., groups with only one feature) were excluded from this evaluation as the univariate and multivariate methods would give the same result.

All features are tested individually with the univariate tests, corrected for multiple-testing with Benjamini–Yekutieli procedure (Benjamini and Yekutieli, 2001) within each compound spectrum, and all compound spectra are tested with the multivariate tests.

For the comparison on the feature level, each feature in a compound spectrum that is classified as differential by the multivariate method is defined as a differential feature.

For different effects and test methods, all features are classified whether they are differential or not, and a confusion matrix can be constructed consisting of the number of true positives (TP), true negatives (TN), false positives (FP), and false negatives (FN). These can be combined into sensitivity, specificity, false positive rate (FPR), and false negative rate (FNR). Repeating the prediction with different thresholds influence the performance, which can be visualized as receiver-operator curves (ROC) and summarized by the area under curve (AUC). The use of ROC curves in metabolomics is also demonstrated in Broadhurst and Kell (2006).

Finally, the evaluation can take place on the level of compound spectra (or metabolites) instead of the feature level and so compares different spectra-wise analysis approaches. This requires the definition how to interpret the multiple individual univariate decisions for a given compound spectrum. Here, all compound spectra where at least one feature was classified as differential by the univariate tests were defined as differential compound spectra. In essence, this is a two-step approach where a test on all univariate *p*-values is performed for each compound spectrum. So on the compound spectra level we can only compare the different spectra-wise analysis approaches, the two multivariate methods, which group intrinsically and the two-step approach, which uses the univariate method as the first step for spectra-wise analysis.

## 3 Results and Discussion

This section covers first an example for the detection of differences between a wildtype and mutant genotype experiment. Then, the analysis of the semi-synthetic ground truth dataset allows an evaluation of the statistical methods with regard to sensitivity, specificity, and area under ROC curves for multiple effects.

### 3.1 Analysis of an experiment with wildtype and mutant plants

First, a real dataset is analyzed. One sample class is comprised of seven *A. th*. Col-0 wildtype plants and a second class of seven samples of an *A. th*. over-expression line, a transposon based activation tagged *A. th*, line from the TAMARA population. Here, the real effect is unknown, and only a few exemplary results are described.

The data processing of the 14 samples results in a 2110 × 14 feature matrix, where CAMERA detected 335 compound spectra. The spectra with just a single feature are excluded from this comparison since the results are identical for both statistical analyses. 28% of all compound spectra have only one feature. The remaining 72% were analyzed with the both univariate and multivariate methods, except for one group with 126 features resulting from the injection peak at the beginning of the chromatography. Overall, 1891 features in 241 feature groups were analyzed.

Table 1 shows two selected compound spectra of an extended diffreport with the two compound spectra no. 40 and no. 82, the univariate *p*-value p.uni for each feature and the multivariate diagonal James *p*-value p.multi for each compound spectrum. The diagonal James test is used because of the small samples size (much smaller than the compound spectra sizes) and the assumed unequal covariance matrices between the two classes.

**Figure 2:**
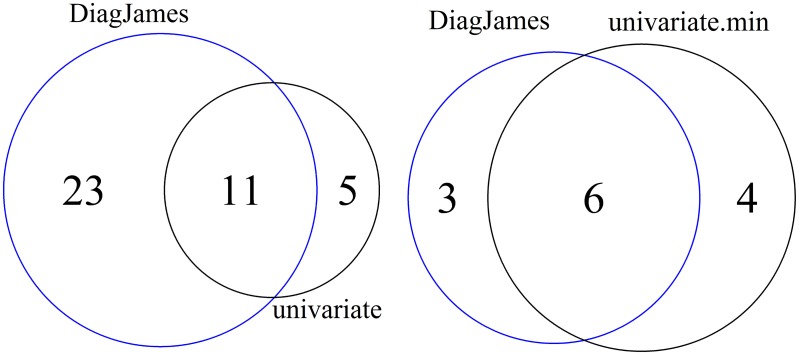
**Venn diagram of differential features and compound spectra in the wildtype-mutant experiment for the significance level of α = 0.01**. Left: number of *features* detected by univariate and multivariate method. Right: number of *compound spectra* detected by the multivariate method, compared to the number of compound spectra where at least one feature was detected univariately.

As shown in Figure 2 (left), 5 features are reported exclusively by the univariate method, while the multivariate approach detected 23 features exclusively, both at a significance level of α = 0.01.

At the compound spectra level, Figure 2 (right) shows that 3 groups are found exclusively by the multivariate approach, which corresponds to case D in Figure 1. All 3 compound spectra found only by the multivariate method are compound spectra with only two or three features.

On the other hand, 4 compound spectra (one of them is a small group with only 2 features, the others have a size of 15, 17, and 35) are found that were not differential in the multivariate test, but where at least one feature was detected by the univariate approach. This corresponds to either case C where all individual features were differential, or case B where only some features were differential. Here, all 4 compound spectra were of type B.

An underlying assumption is the correctness of the CAMERA groupings, where each metabolite corresponds to one compound spectrum. In reality, it can happen that features from one metabolite are split into two (or more) compound spectra. In this case, the multivariate approach looses power, and in the extreme case where a metabolite is split into many singleton spectra achieves the same results as the univariate approach. The opposite case, where two or more metabolites end up in the same compound spectrum can also have a negative influence. If, for example, a differential and a non-differential metabolite are joined, the combined “differentiality” could turn out non-significant and hide one of them.

In this experiment, the biological truth, i.e., which metabolites and features are affected by the over-expression construct is not known. For an objective evaluation, we created a semi-synthetic dataset with simulated fixed effects.

### 3.2 Evaluation with multiple simulated fixed effects

In this second experiment, the performance of the three statistical analysis – univariate and multivariate with both Hotellings-*T*^2^ and the diagonal Hotellings-*T*^2^ – was compared on a dataset of metabolite profiles from *Arabidopsis thaliana*. The xcms processing results in a matrix of 1476 features, and the CAMERA grouping reveals 282 compound spectra. As explained above, for the simulation of the “mutant” class, only a subset of 153 compound spectra with 12 or less features can be used for the ground truth.

We combined the negative set (effect 0.0) with 686 features in 153 compound spectra with the positive set consisting of the same 686 features but with the added effect. For each effect, between 0.0 and 1.4, the final ground truth dataset thus contained 306 compound spectra with a total of 1372 features.

The following exemplifies the results for the fixed effect of 0.5, corresponding to a fold change of ≈1.5 in the original, non-logarithmic data.

For a significance level of α = 0.05, Table 2 shows the summary of the confusion matrix for all three approaches. The multivariate approaches clearly achieve both a better sensitivity and FNR.

**Table 2:** **Comparison of performance of univariate and multivariate tests for a simulated effect of 0.5 and significance level of α = 0.05**.

Method	FP (FPR)	FN (FNR)	TP (sensitivity)	TN (specificity)
Univariate	0 (0%)	427 (62.2%)	259 (37.8%)	686 (100%)
*T*^2^	36 (5.2%)	151 (22%)	535 (78%)	650 (94.8%)
Diag*T*^2^	5 (0.7%)	180 (26.2%)	506 (73.8%)	681 (99.3%)

**Figure 3:**
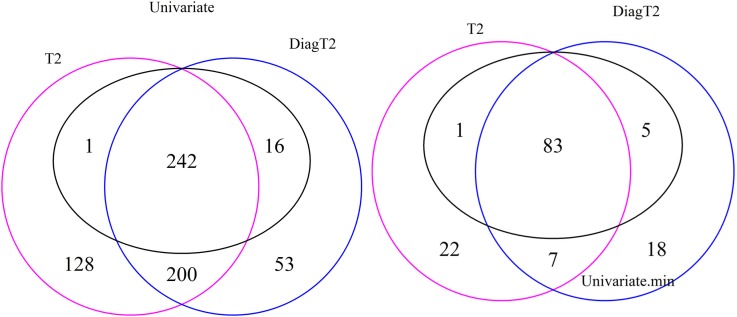
**Venn diagram of differential features and compound spectra in the simulation experiment for the simulated effect 0.5 and significance level of α = 0.05**. Left: number of *features* detected by univariate and multivariate method. Right: number of *compound spectra* detected by the multivariate method, compared to the number of compound spectra where at least one feature was detected univariately.

The Venn diagram in Figure 3 (left) shows the 242 features are detected as differential by all three tests, 243 by both the univariate and the *T*^2^ and 258 by both the univariate and the diagonal *T*^2^. The Comparison of the univariate and the original *T*^2^ shows that 16 features are found only by the univariate and 328 features only by the multivariate method. The same for the diagonal *T*^2^ shows that only 1 feature is found only by the univariate and 253 features only by the multivariate method. Furthermore, 200 features are found by both multivariate methods. It is shown that the feature detection has more overlap between the two multivariate methods than between one of these with the univariate approach. Now, we are especially interested in cases where the multivariate methods identify compound spectra as differential, while the univariate method detects none of the features in the spectra, or cases where the univariate method detects features whose associated compound spectra are missed by the multivariate methods (Figure 3 right). Here, only 7 compound spectra are detected by both multivariate methods, 29 by the original multivariate *T*^2^ and 25 by the diagonal multivariate method, where any feature of this spectra is detected by univariate method. In contrast, 5 compound spectra have at least one feature, which is detected by the univariate test, but the compound spectra itself are not identified by the multivariate *T*^2^ method and 1 compound spectrum in comparison with the diagonal multivariate *T*^2^. 83 groups are detected by all three tests, 84 by univariate and *T*^2^, 98 by univariate and diagonal *T*^2^ (Figure 3 right).

The ROC curve of the three feature detection approaches for a specific effect of 0.5 (Figure S6 in Supplementary Material II) shows the sensitivity and specificity for significance thresholds other than α = 0.05, and confirms that the multivariate method has a higher AUC.

**Figure 4:**
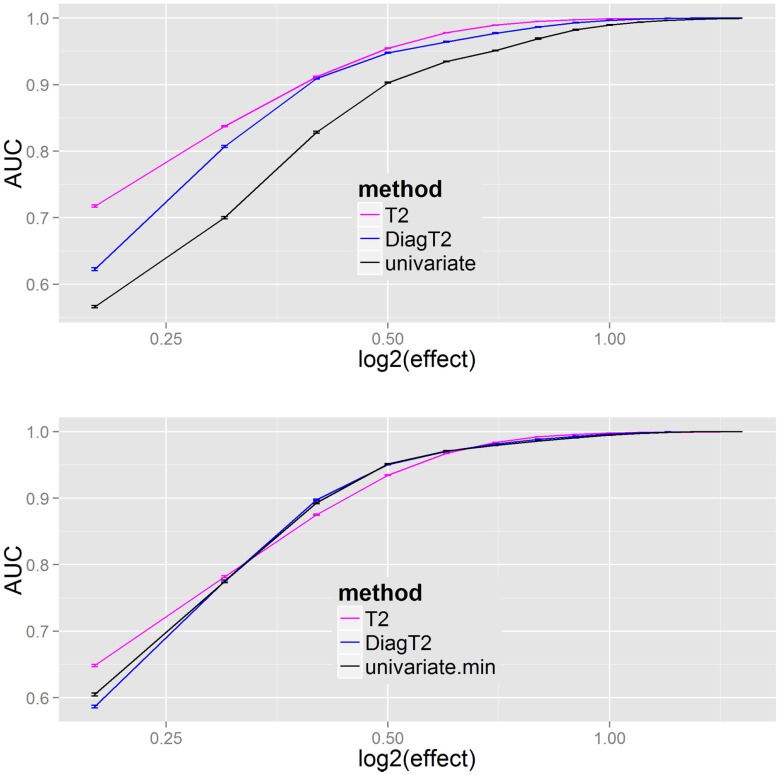
**Results of univariate and multivariate methods in feature detection are compared on the feature level (upper)**. At the compound spectra level (lower) the results of different grouping analysis approaches are shown. For each simulation step, several added effects of 0.2, 0.3, …, 1.4, 1.5 on the “mutant” class, the mean and SE of the evaluated AUCs (results from 100 repetitions) are plotted.

The next question was the behavior of the methods for different effects. The AUC was used as a summary metric of the performance. Figure 4 shows that the multivariate *T*^2^ as well as the diagonal *T*^2^ method has a better AUC for the feature detection compared to the univariate approach for all effects of 0.2, 0.3, …, 1.4, 1.5. To improve the generalization, the sampling of the “mutant” data was repeated 100 times for each effect. Especially for smaller effects, the benefit of the multivariate approach is visible and also that the simplified diagonal *T*^2^ approximates to the original *T*^2^ for larger effects.

The results Figure 4 (bottom) show no particular differences between the different compound spectra level (or grouping) approaches, thus the main benefit results from a joint analysis of compound spectra, while less differences are observed between the joint analysis methods. In Supplementary Material I all methods including James and diagonal James are compared in Figure 2 on the feature level, and Figure 3 on the compound spectra level. In a repeated sensitivity analysis (Supplementary Material III) we show that for small effects and large compound spectra Hotelling’s-*T*^2^ has an advantage over the other grouping approaches.

## 4 Conclusions

In mass spectrometry-based metabolomics data, metabolites (which are the objects of biological interest) will usually give rise to multiple spectral features. In recent years, methods were developed to group these related features into compound spectra. However, the statistical analysis was still based in either individual univariate tests or global multivariate analysis.

We have extended the feature-wise univariate statistic tests to a compound spectra-wise analysis. Using traditional multivariate hypothesis tests, like the Hotelling’s *T*^2^ or James test, the confidence interval becomes a multidimensional ellipsoid that resembles the joint probability for metabolites to be differential more realistically.

On real data of a comparative wildtype-mutant experiment design, the results of the univariate and multivariate tests have an overlap, while some features are detected exclusively by the univariate or multivariate test.

On the synthetic data where the actual effect was known, on the feature level, the resulting AUCs for the multivariate analysis of compound spectra were better than in the univariate case, we recommend to analyze the data compound spectra-wise for biomarker discovery in mass spectrometry metabolomics data. On the compound spectra level the advantage of *T*^2^ over the other spectra-wise approaches is most prominent for noisy data and/or if very small effects should be detectable.

If the CAMERA grouping erronously splits a metabolite into several compound spectra the results of all spectra-wise analyses will approach the multivariate results, and false negatives can occur if a differential and a non-differential metabolite are joined by the compound spectra grouping.

While CAMERA was used in this study, the approaches are readily applicable to any data where individual features from a metabolite are grouped together. In particular, this should allow the analysis of GC/MS data, where the established data analysis typically relies on deconvoluted spectra or mass spectral tags, and where the selection of quantifier ions would have to be repeated for each sample matrix or sample type. The presented approach does not require the selection of representative ions.

The proposed joint analysis of features of a metabolite group as a spectra-wise analysis is the key idea and bridges an important gap between hypotheses tests on individual features on the one hand, and global multivariate methods, which might be more difficult to interpret on the other.

## Author Contributions

SS performed the metabolomics experiment, DT performed the statistical analysis, SN and IG supervised the work. All authors contributed to the manuscript.

## Conflict of Interest Statement

The authors declare that the research was conducted in the absence of any commercial or financial relationships that could be construed as a potential conflict of interest.

## Supplementary Material

This article is accompanied by the following supplemental information which can be found online at http://journal.frontiersin.org/article/10.3389/fbioe.2015.00129:

Presentation 1 **The Supplementary Material I file contains details about the mass spectrometry setup and data processing (Section 1), additional results of the simulation experiment (Section 2), and moreover the formula of the statistical tests (Section 3)**.

Presentation 2 **The Supplementary Material II file shows the ROC curves for the simulation experiment**.

Presentation 3 **The Supplementary Material III file shows repeated figures of the simulation experiment for different datasets**.

Data Sheet 1 **The raw data to the article is available from the MetaboLights repository as accession MTBLS74^2^ and MTBLS169^3^**. The provided *R*-functions in file multivariateDiffreport.R and a vignette file MTBLS169analysis.Rnw is provided, which contains an example analysis on the dataset seven measurements of an *Arabidopsis thaliana* versus 7 measurements of the over-expression line. The Rdata object MTBLS169.Rdata contains the preprocessed MS peak lists and annotations.

## Funding

SS was partially funded by the BMBF (GABIProTect 0315051C). SN acknowledges funding from DFG grant NE/1396/5-1.

## Footnotes

^1^http://www.labman.co.uk/portfolio-type/ipb-cryogenic-grinder-and-feeder-system

^2^http://www.ebi.ac.uk/metabolights/MTBLS74

^3^http://www.ebi.ac.uk/metabolights/MTBLS169
