# Supplementary material for: Joint Analysis of Dependent Features within Compound Spectra Can Improve Detection of Differential Features
Source: Front Bioeng Biotechnol. 2015 Sep 24;3:129. doi: 10.3389/fbioe.2015.00129 (PMC4585098; doi:10.3389/fbioe.2015.00129)
Supplement: Presentation 1 — The Supplementary Material I file contains details about the mass spectrometry setup and data processing (Section 1), additional results of the simulation experiment (Section 2), and moreover the formula of the statistical tests (Section 3). [file Presentation_1.PDF]

Frontiers in Bioengineering

Research Article
201519 July 2015

Supplementary Material I: Joint analysis of dependent features within compound spectra can improve detection of differential features

Diana Trutschel [1,2], Stephan Schmidt [1], Ivo Grosse [2,3] and Steffen Neumann [1,*]

[1] *Leibniz Institute of Plant Biochemistry, Department of Stress and Developmental Biology, Halle, Germany*
[2] *Martin-Luther-University Halle-Wittenberg, Institute of Computer Science, Halle, Germany*
[3] *German Centre for Integrative Biodiversity Research (iDiv) Halle-Jena-Leipzig, Leipzig, Germany*

Correspondence*:
Steffen Neumann
Leibniz Institute of Plant Biochemistry, Department of Stress and Developmental Biology, Weinberg 3, 06120 Halle, Germany, sneumann@ipb-halle.de

1 SUPPLEMENTARY DATA: SAMPLE PREPARATION AND METABOLITE PROFILING PROTOCOLS

The following are the protocols reported for the Metabolights datasets MTBLS74 and MTBLS169.

Growth conditions Perceval growth chamber, short day conditions - light: 9:00-17:00, temperature day/night 23/23 °C

Sample collection Leafs were harvested and processed with a cryogrinding robot: Fill 50% volume of the scintillation vials with frozen leaf material (leafs can be broken to fit in). Add 3 steel balls, diameter 5 mm. Use robot sample program Arabidopsis leaf. Weights: 100 mg weight, 2 mg tolerance.

Extraction To the frozen leaf material 200 μL methanol/water, 80/20 (v/v) pre-cooled at 40°C and spiked with biochanin A (5 μM, Sigma) as internal standard, the samples were immediately vortexed for 15 s. Probes were placed in ice to slowly warm up to 0°C. Then the probes were vortexed, sonicated for 15 min at 20°C and centrifuged for 10 min at 19000 /g/. The supernatants were transferred to new 2-ml tubes and the remaining pellets subjected to a second extraction using 200 μL methanol/water, 80/20 (v/v). The combined extracts were evaporated to dryness in a vacuum centrifuge at 30°C, reconstituted in 200 μL methanol/water, 30/70 (v/v), vortexed and centrifuged for 10 min at 19000 /g/. The supernatants were transferred to glass vial for measurement.

Chromatography Chromatographic separations were performed on an Acquity UPLC system (Waters) equipped with [ADDED:] a reverse phase C18 column HSS T3 (100 x 1.0 mm, particle size 1.8 μm, Waters, oven temperature 40 °C) applying the following binary gradient at a flow rate of 150 μL/min: 0-1 min, isocratic 95% A (water/formic acid, 99.9/0.1 (v/v)), 5% B (acetonitrile/formic acid, 99.9/0.1 (v/v)); 1-16 min, linear from 5 to 45% B; 16-18 min, isocratic 95% B; 18-20 min, isocratic 5% B. The injection volume was 3.0 μL (full loop injection).

Mass spectrometry Eluting compounds were detected from m/z 100-1000 using a micrOTOF-Q hybrid quadrupole time-of-flight mass spectrometer (Bruker Daltonics) equipped with an Apollo II electro-spray ion source in positive and negative ion mode using following instrument settings: nebulizer gas, nitrogen, 1.6 bar; dry gas, nitrogen, 6 L/min, 190°C; capillary, 4500 V; end plate offset, -500 V; funnel 1 RF, 200 V; funnel 2 RF, 200 V; in-source CID energy, 0 V; hexapole RF, 100 V; quadrupole ion energy, 5 eV; collision gas, nitrogen; collision energy, 7 eV; collision RF 150/350 V (timing 50/50); transfer time, 70 μs; pre pulse storage, 5 μs; pulser frequency, 10 kHz; spectra rate, 3 Hz. Mass spectra were acquired in centroid mode. Mass calibration of individual raw data files was performed on lithium formate cluster ions obtained by automatic infusion of 20 μL 10 mM lithium hydroxide in isopropanol/water/formic acid, 49.9/49.9/0.2 (v/v/v) at a gradient time of 18 min using a diverter valve.

Data transformation Raw data files were converted to mzData format using the vendor-specific CompassXport (http://www.bdal.de/) with useRecalibratedSpectra=TRUE and processed using the XCMS package [**?**] [1]

XCMS settings for processing LC/MS data were prefilter=3,100; snthr=6; ppm=30, peakwidth=5,10. For alignment group.density function with parameters mzwid=0.015, minfrac=1 and bw=3 was used. For all samples we create a dummy variable in phenoData to achieve a full data matrix with no unobserved intensities and prevent the using of `fillPeaks()`, a function to impute missing values. Up to the end all extracted feature intensities were logarithmized to ensure approximately normal distributed intensities (see all in Program 1).

Feature grouping to compound spectra The rows in the matrix are annotated and the grouping of related features into compound spectra was performed, both with the package CAMERA[**?**]. At first the function `xsAnnotate()` with all samples, then for grouping the function `groupFWHM()` was used (see all in Program 1). Figure 1 shows, that the size of the identified compound spectra varies from 1 to 123, although it has been noted that the compound spectra of size 123 is an injection peak and would not be analysed.

Program 1 The R-code for data transformation to create feature intensities of MS experiment and feature grouping to compound spectra for the *Arabidopsis thaliana* plant experiment.

```
xset<-xcmsSet(mzfiles,
 method="centWave",ppm=30,peakwidth=c(5,10),snthresh=6)
#save the orign classification
origPhenoData <- phenoData(xset)
#set a dummy variable for all samples
phenoData(xset) <- rep("dummy", times=length(origPhenoData[,1]))
#grouping features over all samples
xset<-group(xset, minfrac=1, bw=3,mzwid=0.015)
#set the old classification
phenoData(xset) <- origPhenoData
#logaritmize the feature intensities
vals<-log(groupval(xset, value="into",  "medret"), base=2)
#use CAMERA to annotate feature groups
an <- xsAnnotate(xset,sample=seq(1,length(sampnames(xset))))
# group the peak data after the FWHM of the retention time
an <- groupFWHM(an)
#identified features to each feature group
groupofpeaks<-an@pspectra
```

[1] `http://bioconductor.org/packages/release/bioc/html/xcms.html`

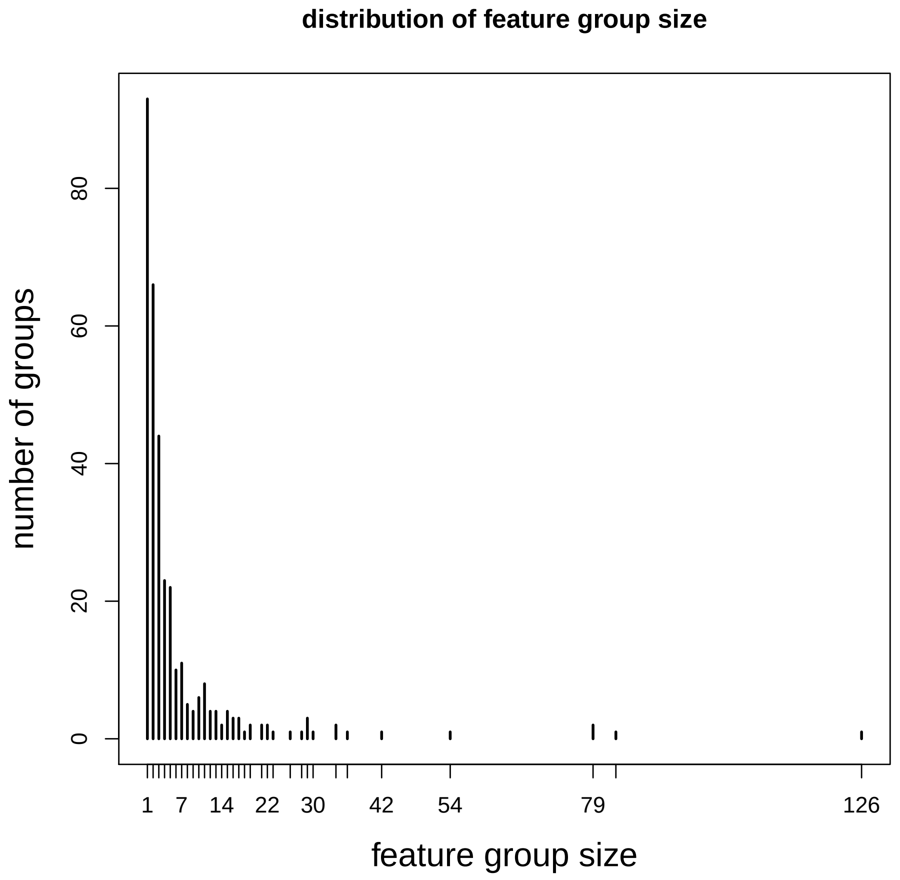

Figure 1. Distribution of size of compound spectra identified with CAMERA annotation for the wildtype-mutant experiment.

2 EVALUATION WITH MULTIPLE SIMULATED FIXED EFFECTS

2.1 ALGORITHM FOR THE SIMULATION

3 MATHEMATICAL DETAILS

Table 1 mathematical formulas of all proposed univariate and multivariate tests are given.

REFERENCES

[1]Frederick Mosteller and R. A. Fisher. Questions and Answers. *The American Statistician*, 2(2):30–31, October 1948.

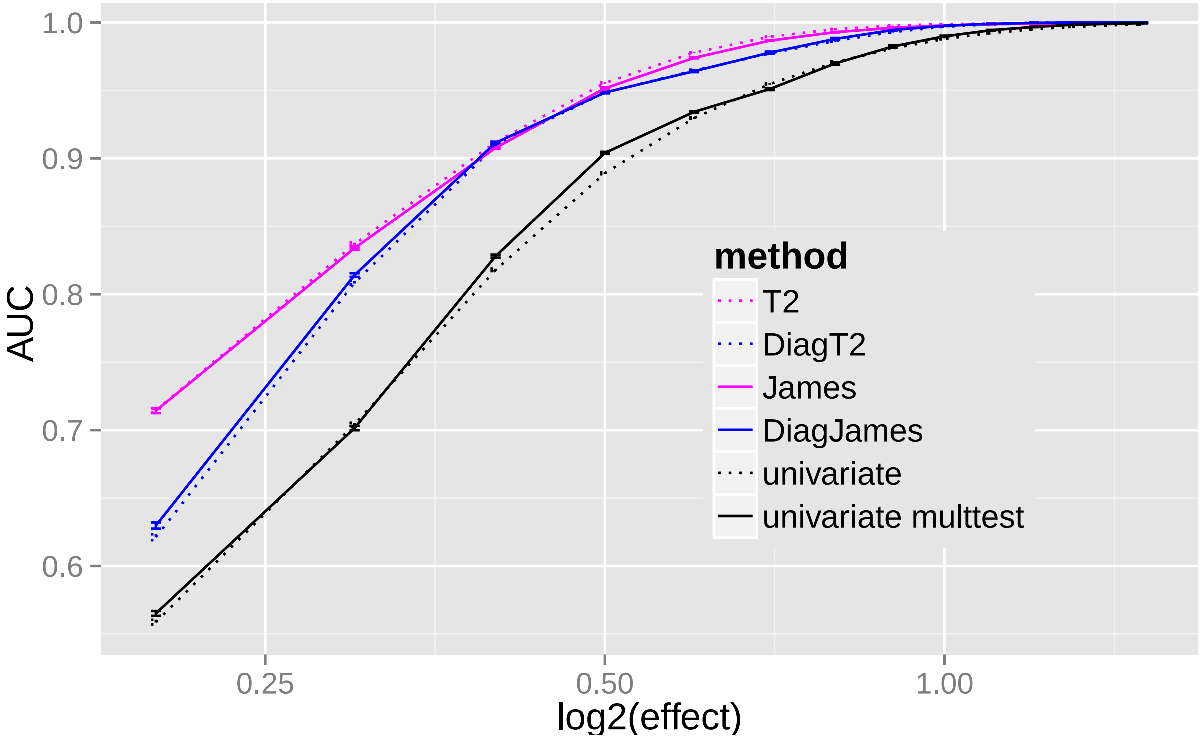

Figure 2. Compare results of univariate and multivariate test in feature detection for several effects of $0.2, 0.3, ..., 1.4, 1.5$, on feature level. .

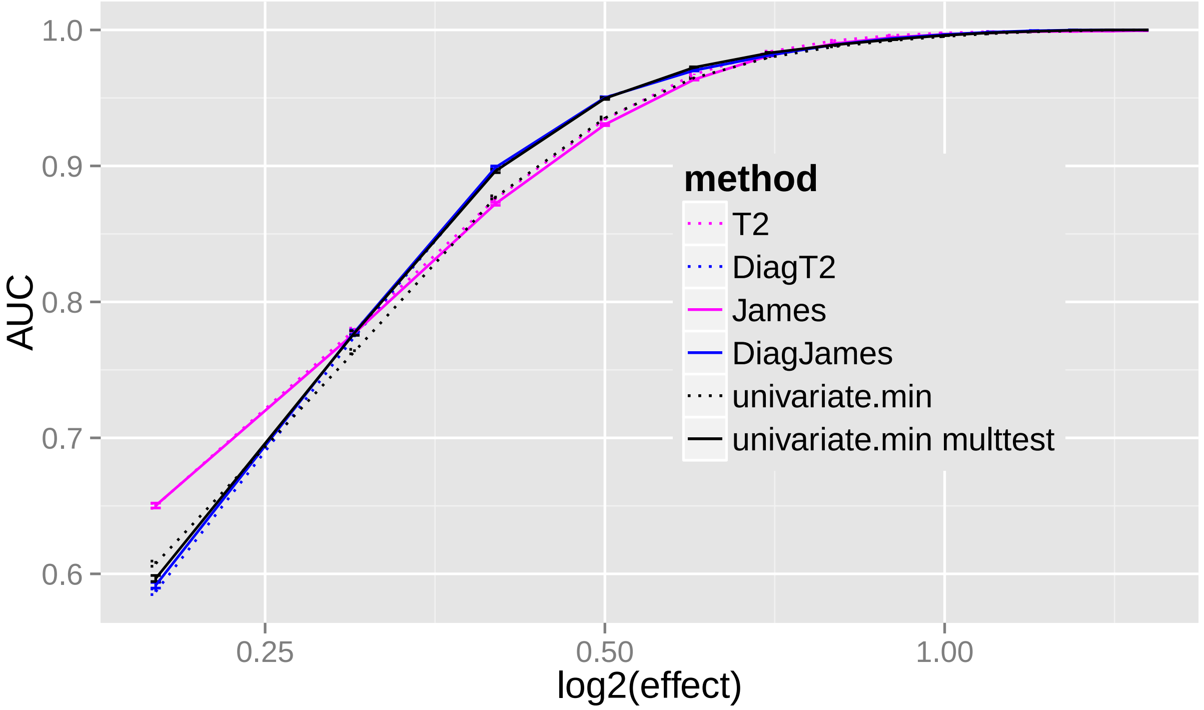

Figure 3. Compare results of different tests on compound spectra detection (different multivariate methods including several grouping methods) for several effects of $0.2, 0.3, ..., 1.4, 1.5$. .

Algorithm 1 Simulation of a gold-standard dataset on a real dataset to determine the quality of univariate and multivariate tests to detect differentiell features or compound spectra for several numbers effects eff_i.

```
INPUT: DS = dataset of MS measurements of plants of one genotype
INPUT: CSA = compound spectra annotation corresponding to the DS
F #number of row of DS (number of features)
N #number of columns of DS (number of samples)
C #number of compound spectra
# split DS into two classes
for DS do
    class WT ← DS[,1 : N/2] # 1 : N/2 samples of DS
    class MU.ref ← DS[,N/2 + 1 : N] # N/2 + 1 : N samples of DS
    for all compound spectra j=1,..,C do
        estimate covariance matrix Σ_j out of MU.ref
for all eff ∈ c(0.2, 0.3, ..., 1.5) do
    while iter < 1000 do
        for all compound spectrum j ∈ 1, ..., C do
            # combine Negative dataset as all samples came from the same genotype
            MU(j) ← Mu.ref(j) + X(j),
            X(j)∼ N(0, σ_j) for each compound spectra j, j=1,..,C
            class MU←c(MU(1),...,MU(C))
            Negatives ← c(WT, MU)
            calculate p.Hotellings, p.DiagHottellings
            for ∀ feature k ∈ 1, ..., K_c within compound spectrum j do
                calculate p.univariate
                overtaken p.Hotellings, p.DiagHottellings
                if p < 0.05 then
                    FP
                else
                    TN
            # combine Positive dataset as an effect is added in one class
            MU(j) ← Mu.ref(j) + X(j),
            X(j)∼ N(eff, σ_j)for each compound spectra j, j=1,..,C
            class MU ←c(MU(1),...,MU(C))
            Positives ← c(WT, MU)
            calculate p.Hotellings, p.DiagHottellings
            for ∀ feature k ∈ 1, ..., K_c within compound spectrum j do
                calculate p.univariate
                overtaken p.Hotellings, p.DiagHottellings
                if p < 0.05 then
                    TP
                else
                    FN
Calculate the number of TP, TN, FP, FN, AUC of all 1000 repeats
```

Supplementary Table 1. Formula of the univariate Student's t test citeStudent08probableerrorof, Welch's t-test [**?**], the multivariate Hotelling's T^2 test [**?**] in comparison with the James test [**?**] for two sample classes, where n_i number of observations of sample class i. The Welch's t-test is used to compare two univariate samples on difference in means $\bar{x_1}, \bar{x_2}$ with the assumption of unequal variances s_1^2, s_2^2, where ν_1 are the degrees of freedom associated with the variance estimate of sample i. The Hotelling's T^2 test is used compare two p-dimensional samples on difference in mean vectors $\bar{X_1}, \bar{X_2}$ with the assumption of unknown, but equal covariance matrices S_1, S_2, so the the pooled covariance matrix S_p is used creating the test statistic. The multivariate equivalent to the univariate Welch's t-test is the James test. Here, unknown and unequal covariance matrices S_1, S_2 are assumed. The proposed uncorrelated type of James test uses covariance matrices with only the diagonal entries, the variances.

**A.**	univariate<br>Student's t-test	multivariate<br>Hotelling's T^2
test statistic	$t = \frac{\bar{x_1}-\bar{x_2}}{s_p\sqrt{\frac{1}{n_1}+\frac{1}{n_2}}}$<br>$s_p = \frac{(n_1-1)s_1+(n_2-1)s_2}{n_1+n_2-2}$	$T^2 = \frac{n_1 n_2}{n_1+n_2}\left(\bar{X_1}-\bar{X_2}\right)^T S_p^{-1}\left(\bar{X_1}-\bar{X_2}\right),$<br>$S_p = \frac{(n_1-1)S_1+(n_2-1)S_2}{n_1+n_2-2}$
H_0	$t \leq t_{\frac{\alpha}{2},n_1+n_2-2},$	$\frac{(n_1+n_2-p-1)}{(n_1+n_2-2)p}T^2 \leq F_{\alpha,p,n_1+n_2-p-1}$

**B.**	univariate<br>Welch's t-test	multivariate<br>James test
test statistic	$t = \frac{\bar{x_1}-\bar{x_2}}{\sqrt{\frac{s_1^2}{n_1}+\frac{s_2^2}{n_2}}}$	$T_u^2 = \left(\bar{X_1}-\bar{X_2}\right)^T S^{-1}\left(\bar{X_1}-\bar{X_2}\right),$<br>$S = \frac{S_1}{n_1}+\frac{S_2}{n_2}$
H_0	$t \leq t_{\frac{\alpha}{2},\nu},$<br>$\nu \approx \frac{\left(\frac{s_1^2}{n_1}+\frac{s_2^2}{n_2}\right)^2}{\frac{s_1^4}{n_1^2\nu_1}+\frac{s_2^4}{n_2^2\nu_2}}$	$T_u^2 \leq \chi^2_{\alpha,A+B\chi^2_{1-\alpha,p}}$<br>$A = 1+\frac{1}{2p}\sum_{i=1}^{2}\frac{tr(S^{-1}S_i)^2}{n_i-1}$, tr=trace of matrix<br>$B = \frac{1}{p(p+2)}\left[\frac{1}{2}\sum_{i=1}^{2}\frac{tr(S^{-1}S_i)^2}{n_i-1}+\frac{1}{2}\sum_{i=1}^{2}\frac{(trS^{-1}S_i)^2}{n_i-1}\right]$
