# Supplementary material for: Joint Analysis of Dependent Features within Compound Spectra Can Improve Detection of Differential Features
Source: Front Bioeng Biotechnol. 2015 Sep 24;3:129. doi: 10.3389/fbioe.2015.00129 (PMC4585098; doi:10.3389/fbioe.2015.00129)
Supplement: Presentation 2 — The Supplementary Material II file shows the ROC curves for the simulation experiment. [file Presentation_2.PDF]

Supplementary Material II: Joint analysis of dependent features within compound spectra can improve detection of differential features

Diana Trutschel[1], Stephan Schmidt[1], Ivo Grosse[2,3], Steffen Neumann[1]

July 17, 2015

[1]Leibniz Institute of Plant Biochemistry, Department of Stress and Developmental Biology,
Weinberg 3, 06120 Halle, Germany, sneumann@IPB-Halle.DE
[2]Martin-Luther-University Halle-Wittenberg, Institute of Computer Science, Von-Seckendorff-Platz 1, 06120 Halle, Germany
[3] German Centre for Integrative Biodiversity Research (iDiv) Halle-Jena-Leipzig, Leipzig, Germany

1 ROC for each effect

This document provides additional information for the article "Joint analysis of dependent features within compound spectra can improve detection of differential features". Here, we provide for the evaluation experiment in Section 3.1 in the main manuscript the receiver-operator curves (ROC) for each simulated effect of eff $= 0.0, 0.1, ..., 1.4$. The ROC visualise prediction, the sensitivity and specificity, with different thresholds. For each effect one plot shows the ROC for all three methods, the univariate test, the multivariate Hotellings and Diagonal-Hotellings.

ROC for effect = 0

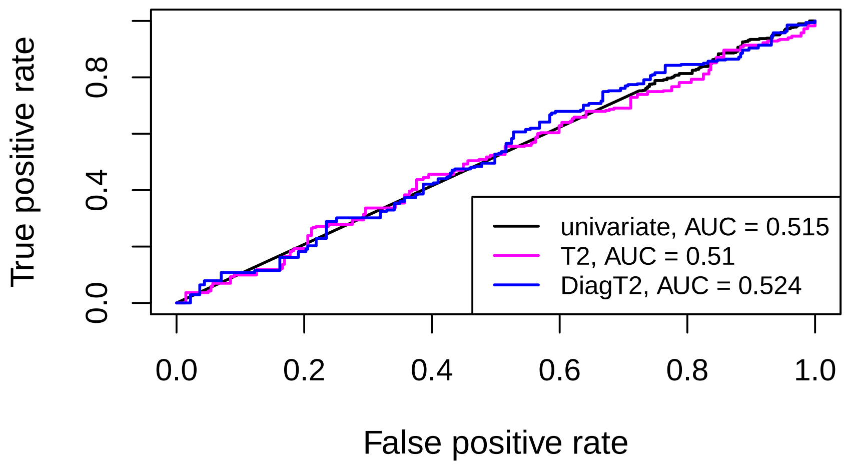

ROC for effect = 0.1

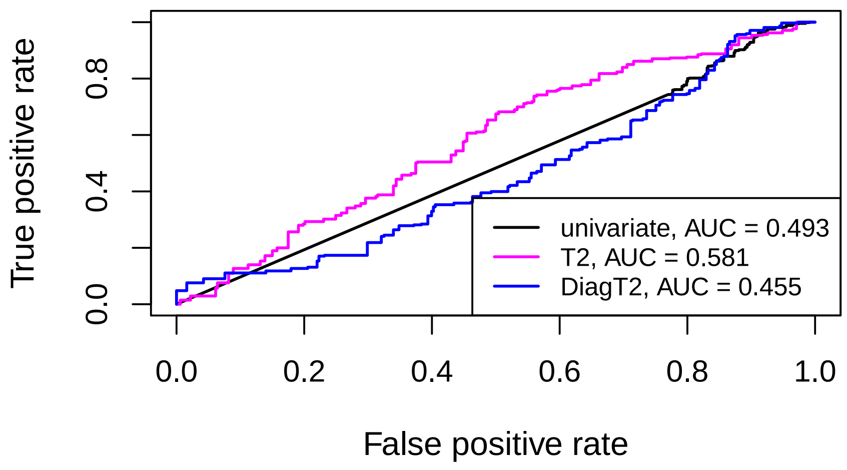

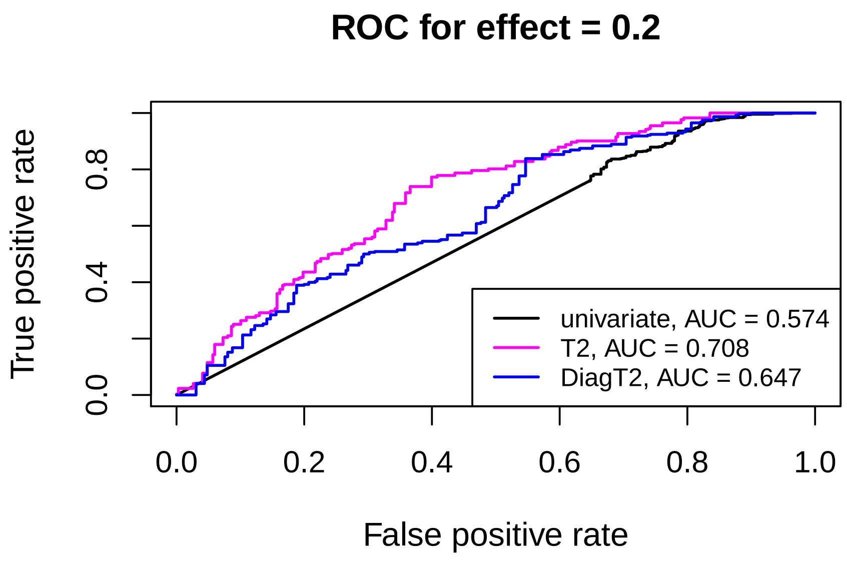
ROC for effect = 0.2
True positive rate
0.0
0.4
0.8
0.0
0.2
0.4
0.6
0.8
1.0
False positive rate
univariate, AUC = 0.574
T2, AUC = 0.708
DiagT2, AUC = 0.647

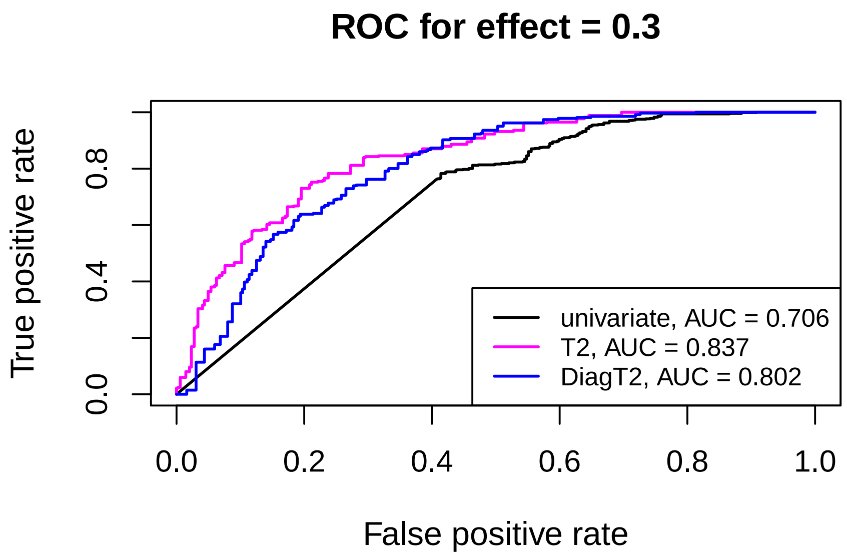
ROC for effect = 0.3
True positive rate
0.0
0.4
0.8
0.0
0.2
0.4
0.6
0.8
1.0
False positive rate
univariate, AUC = 0.706
T2, AUC = 0.837
DiagT2, AUC = 0.802

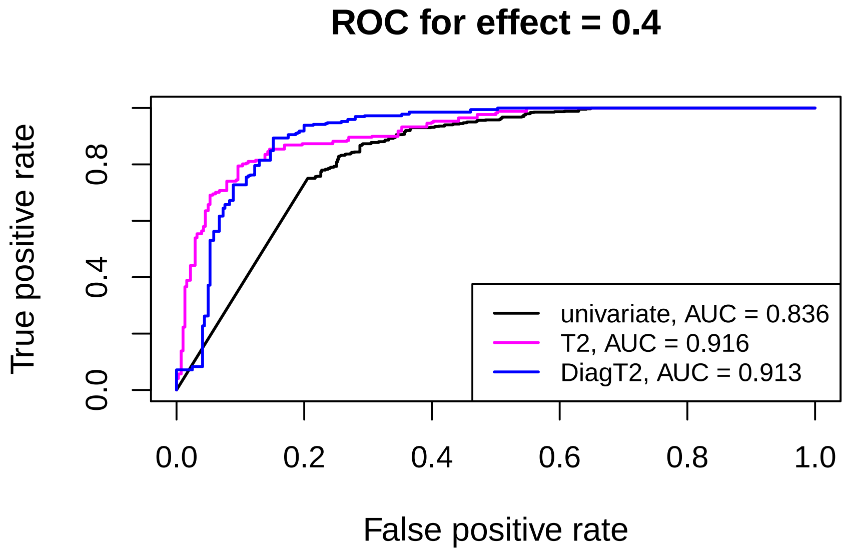
ROC for effect = 0.4
True positive rate
0.0
0.4
0.8
0.0
0.2
0.4
0.6
0.8
1.0
False positive rate
univariate, AUC = 0.836
T2, AUC = 0.916
DiagT2, AUC = 0.913

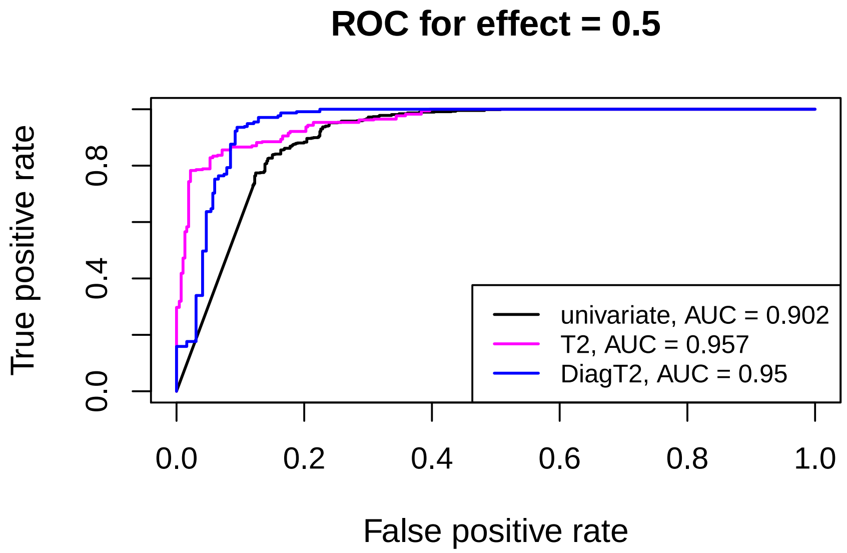
ROC for effect = 0.5
True positive rate
0.0
0.4
0.8
0.0
0.2
0.4
0.6
0.8
1.0
False positive rate
univariate, AUC = 0.902
T2, AUC = 0.957
DiagT2, AUC = 0.95

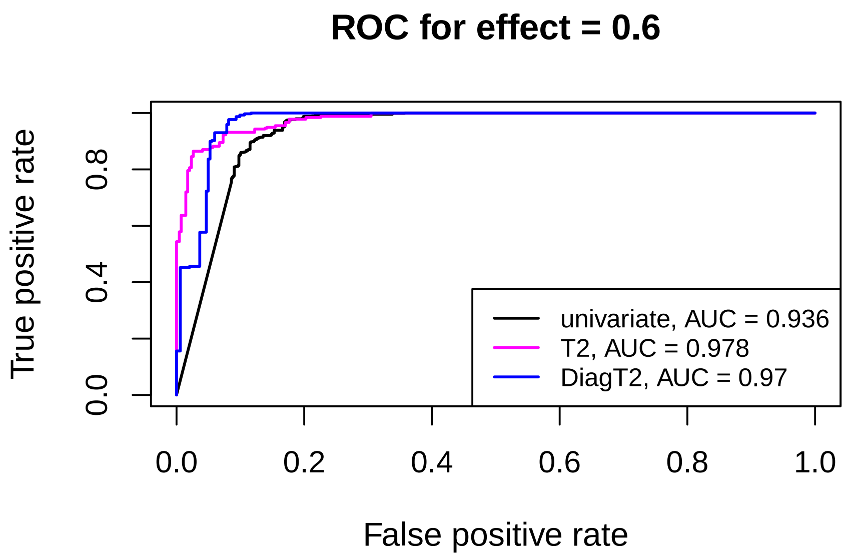
ROC for effect = 0.6
True positive rate
0.0
0.4
0.8
False positive rate
0.0
0.2
0.4
0.6
0.8
1.0
univariate, AUC = 0.936
T2, AUC = 0.978
DiagT2, AUC = 0.97

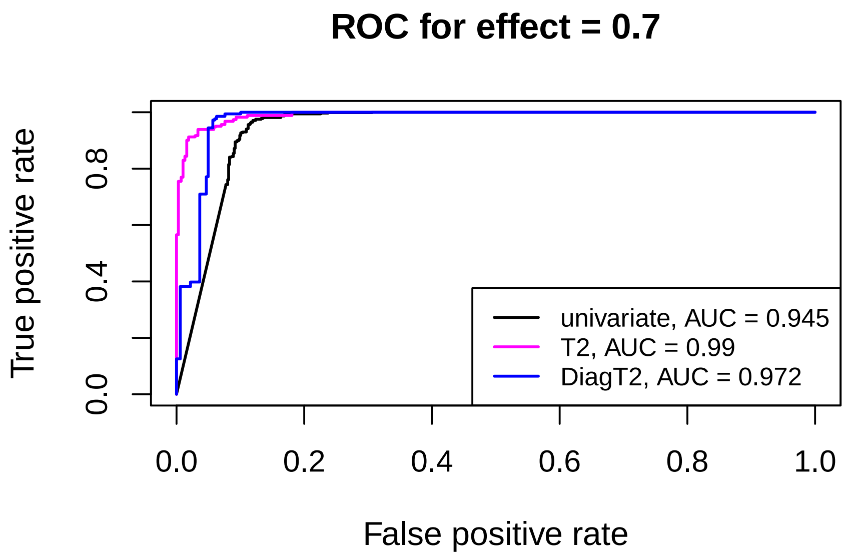
ROC for effect = 0.7
True positive rate
0.0
0.4
0.8
False positive rate
0.0
0.2
0.4
0.6
0.8
1.0
univariate, AUC = 0.945
T2, AUC = 0.99
DiagT2, AUC = 0.972

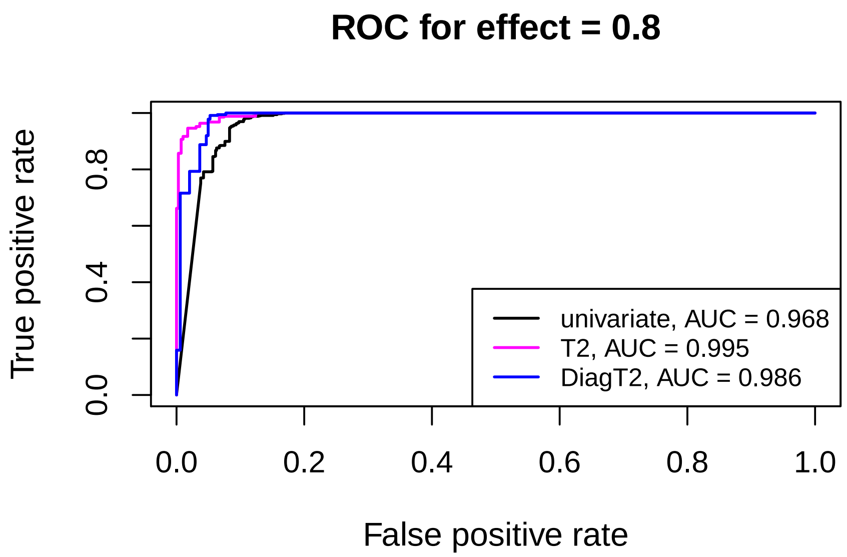

ROC for effect = 0.8
True positive rate
0.0
0.4
0.8
False positive rate
0.0
0.2
0.4
0.6
0.8
1.0
univariate, AUC = 0.968
T2, AUC = 0.995
DiagT2, AUC = 0.986

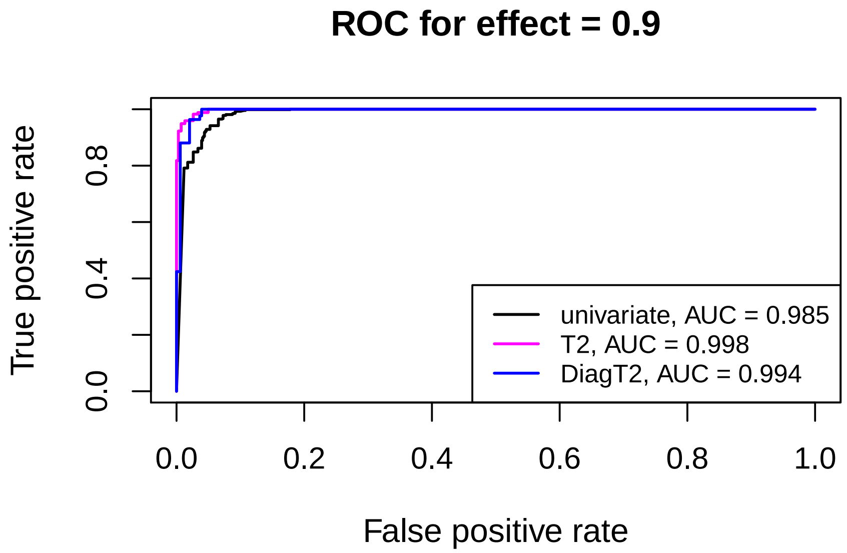

ROC for effect = 0.9
True positive rate
0.0
0.4
0.8
False positive rate
0.0
0.2
0.4
0.6
0.8
1.0
univariate, AUC = 0.985
T2, AUC = 0.998
DiagT2, AUC = 0.994

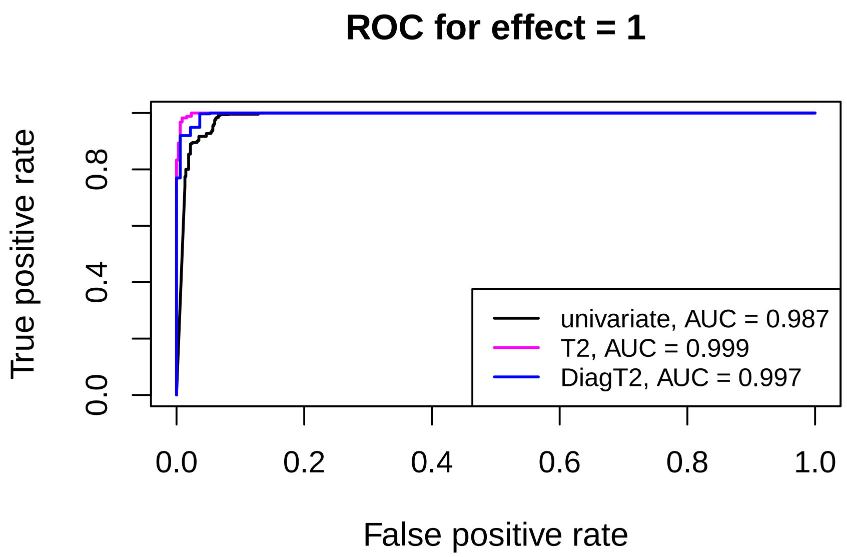
ROC for effect = 1
True positive rate
0.0
0.4
0.8
0.0
0.2
0.4
0.6
0.8
1.0
False positive rate
univariate, AUC = 0.987
T2, AUC = 0.999
DiagT2, AUC = 0.997

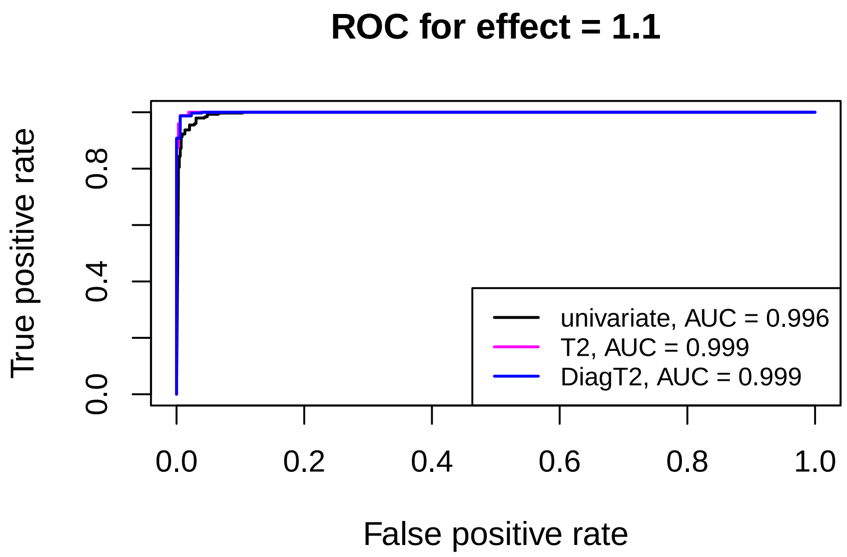
ROC for effect = 1.1
True positive rate
0.0
0.4
0.8
0.0
0.2
0.4
0.6
0.8
1.0
False positive rate
univariate, AUC = 0.996
T2, AUC = 0.999
DiagT2, AUC = 0.999

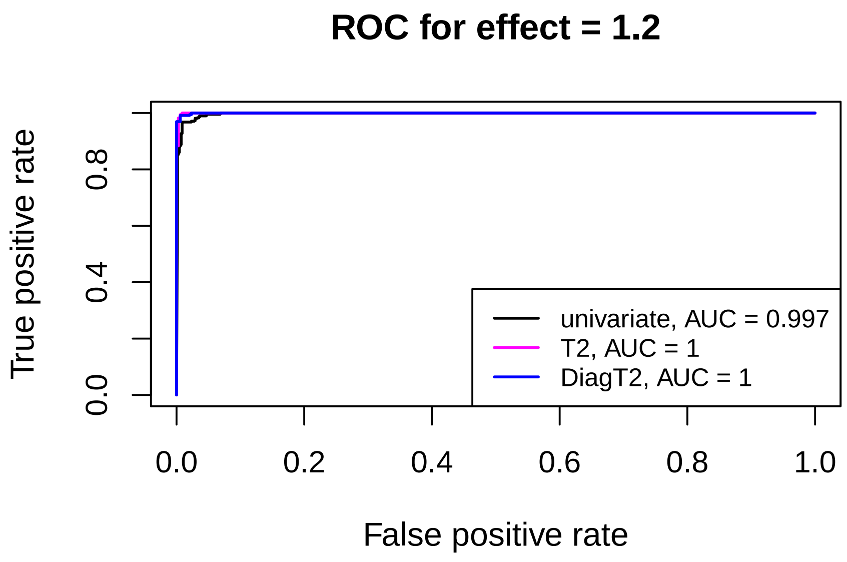
ROC for effect = 1.2
True positive rate
0.0
0.4
0.8
0.0
0.2
0.4
0.6
0.8
1.0
False positive rate
univariate, AUC = 0.997
T2, AUC = 1
DiagT2, AUC = 1

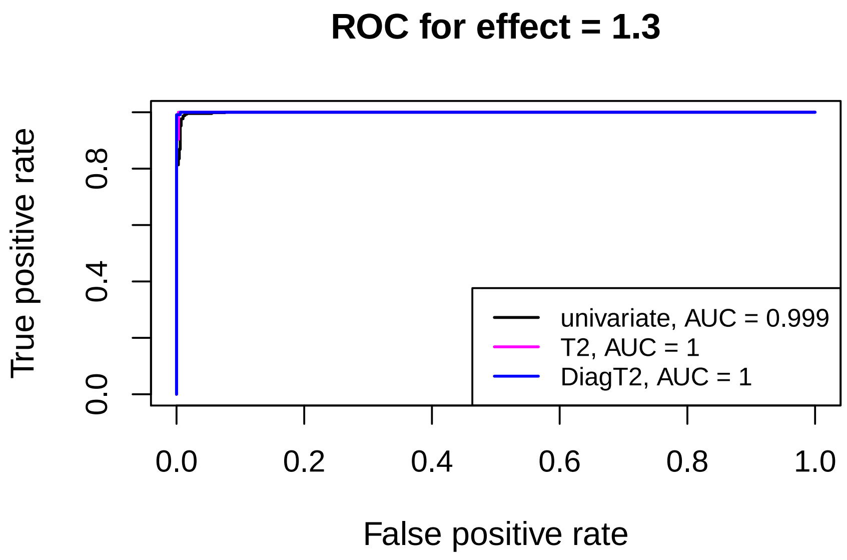
ROC for effect = 1.3
True positive rate
0.0
0.4
0.8
0.0
0.2
0.4
0.6
0.8
1.0
False positive rate
univariate, AUC = 0.999
T2, AUC = 1
DiagT2, AUC = 1

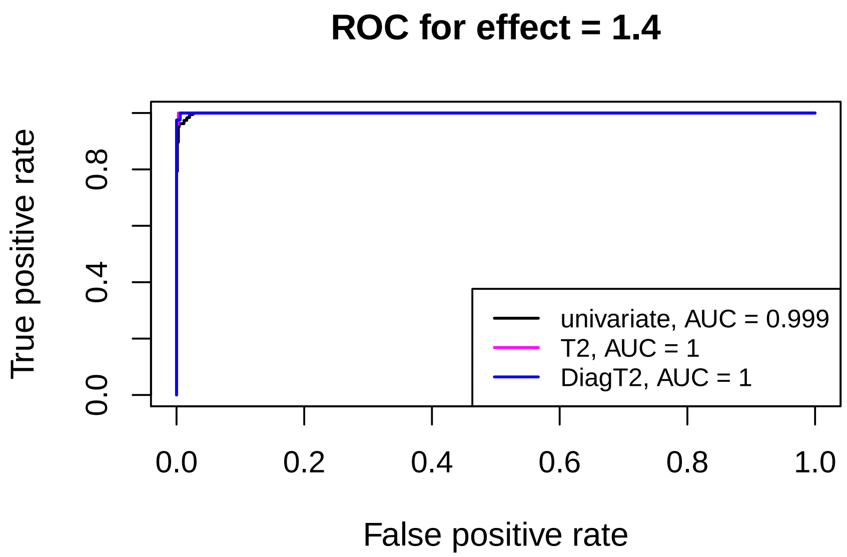
ROC for effect = 1.4
True positive rate
0.0
0.4
0.8
0.0
0.2
0.4
0.6
0.8
1.0
False positive rate
univariate, AUC = 0.999
T2, AUC = 1
DiagT2, AUC = 1
