# Supplementary material for: Joint Analysis of Dependent Features within Compound Spectra Can Improve Detection of Differential Features
Source: Front Bioeng Biotechnol. 2015 Sep 24;3:129. doi: 10.3389/fbioe.2015.00129 (PMC4585098; doi:10.3389/fbioe.2015.00129)
Supplement: Presentation 3 — The Supplementary Material III file shows repeated figures of the simulation experiment for different datasets. [file Presentation_3.PDF]

Supplementary Material III: Joint analysis of dependent features within compound spectra can improve detection of differential features

Diana Trutschel[1,2], Stephan Schmidt[1], Ivo Grosse[2,3], Steffen Neumann[1]

July 17, 2015

[1]Leibniz Institute of Plant Biochemistry, Department of Stress and Developmental Biology,
Weinberg 3, 06120 Halle, Germany, sneumann@IPB-Halle.DE
[2]Martin-Luther-University Halle-Wittenberg, Institute of Computer Science, Von-Seckendorff-Platz 1, 06120 Halle, Germany
[3] German Centre for Integrative Biodiversity Research (iDiv) Halle-Jena-Leipzig, Leipzig, Germany

1 Analysis of different subgroups of full dataset

This document provides additional information for the article "Joint analysis of dependent features within compound spectra can improve detection of differential features".

Here, we provide for the evaluation experiment in Section 3.1 in the main manuscript a repeated analysis (with 50 repeats) for several subsets results on different size of compound spectra.

1. compound spectra of size 2-3 (dataset 1)
2. compound spectra of size 4-6 (dataset 2)
3. compound spectra of size 7-9 (dataset 3)
4. compound spectra of size >9 (dataset 4)

It results in subdatasets with 1) 82, 2) 38, 3) 18, 4) 15 compound spectra including 1) 194, 2) 187, 3) 142, 4) 163 features. Each plot compares results of univariate and multivariate test in feature detection for several effects of $0.2, 0.3, ..., 1.4, 1.5$ with a given significance level of $\alpha = 0.05$. At first for each datasets one figure shows the comparison on feature level, secondly on compound spectra level. For each simulation step the mean and SE of the AUCs of all 100 repeats are plotted.

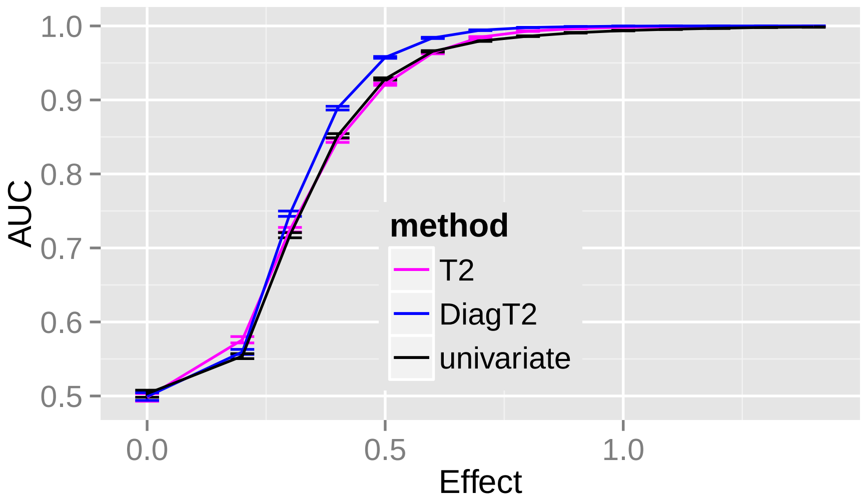

Figure 1: AUC versus effect at feature level for Dataset 1

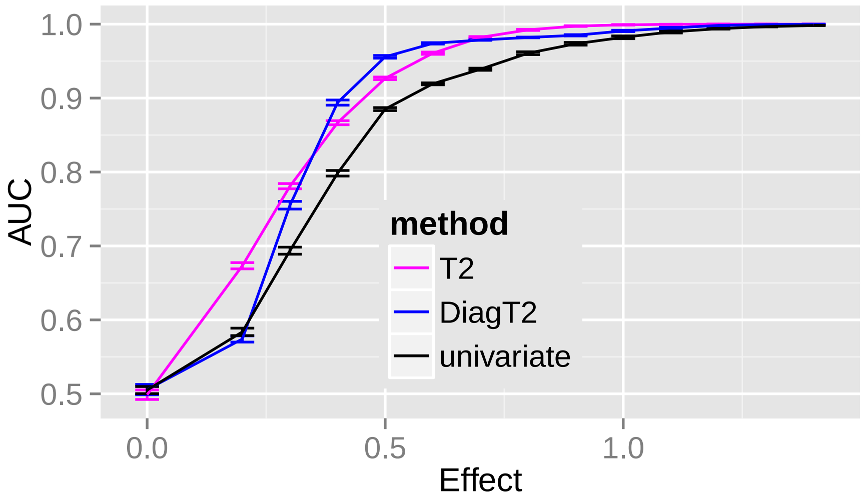

Figure 2: AUC versus effect at feature level for Dataset 2

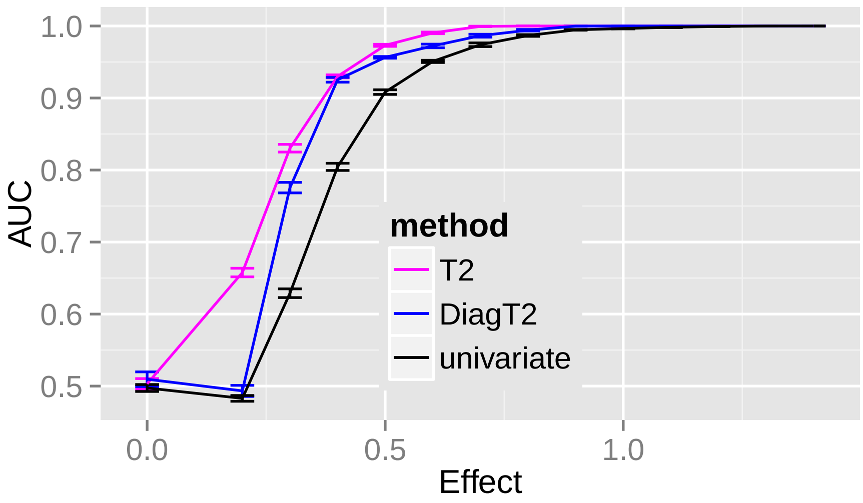

Figure 3: AUC versus effect at feature level for Dataset 3

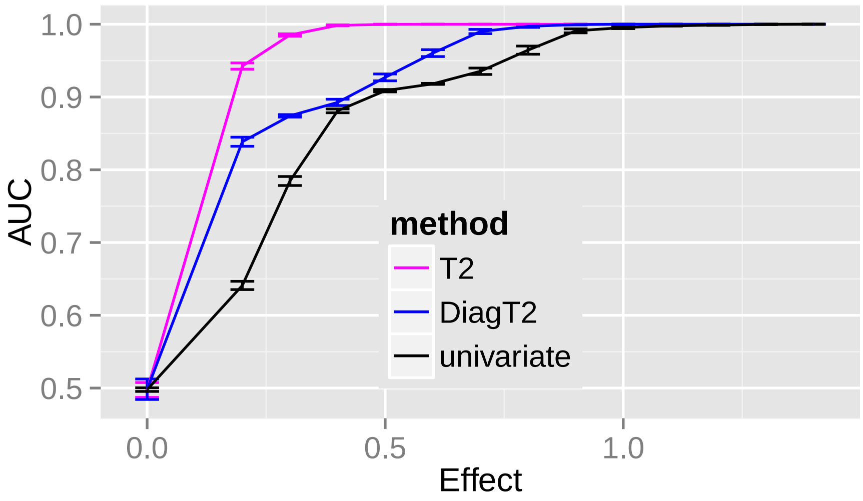

Figure 4: AUC versus effect at feature level for Dataset 4

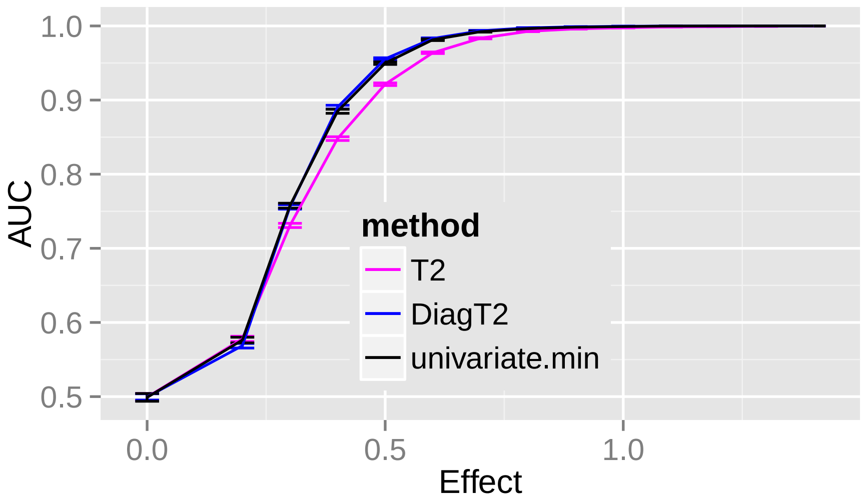

Figure 5: AUC versus effect at compound spectra level for Dataset 1

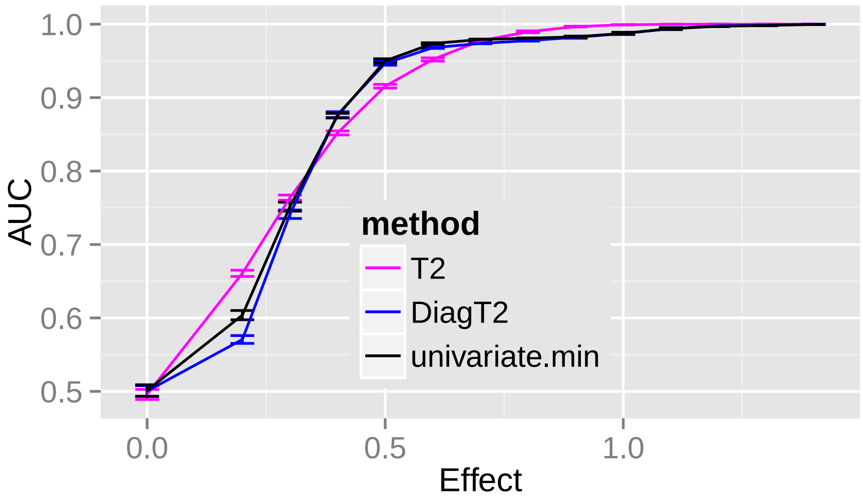

Figure 6: AUC versus effect at compound spectra level for Dataset 2

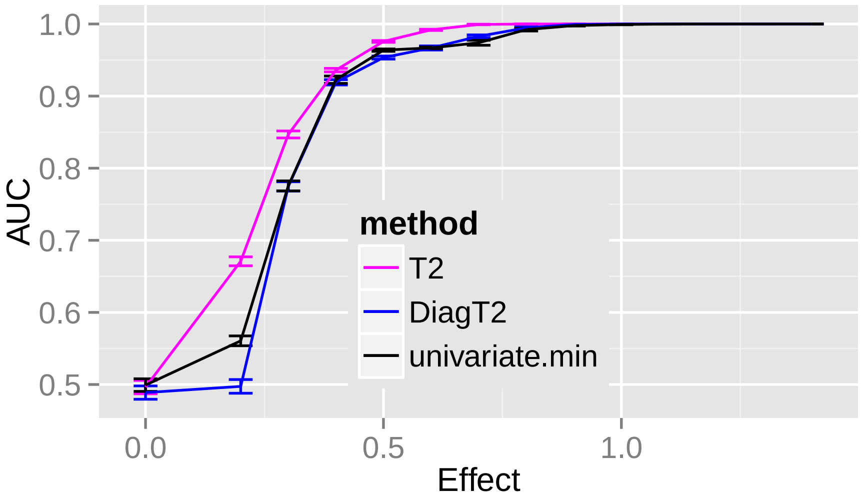

Figure 7: AUC versus effect at compound spectra level for Dataset 3

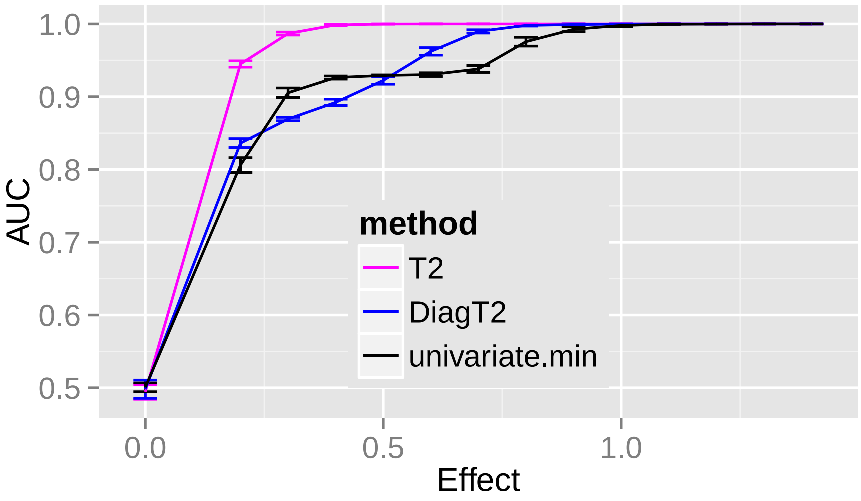

Figure 8: AUC versus effect at compound spectra level for Dataset 4
