# Supplementary material for: Joint Analysis of Dependent Features within Compound Spectra Can Improve Detection of Differential Features
Source: Front Bioeng Biotechnol. 2015 Sep 24;3:129. doi: 10.3389/fbioe.2015.00129 (PMC4585098; doi:10.3389/fbioe.2015.00129)
Supplement: Data Sheet 1 — The raw data to the article is available from the MetaboLights repository as accession MTBLS742 and MTBLS1693. The provided R-functions in file multivariateDiffreport.R and a vignette file MTBLS169analysis.Rnw is provided, which contains an example analysis on the dataset seven measurements of an Arabidopsis thaliana versus 7 measurements of the over-expression line. The Rdata object MTBLS169.Rdata contains the preprocessed MS peak lists and annotations. [file Data_Sheet1.ZIP › MTBLS169analysis.pdf]

Vignette: Multivariate analysis of Arabidopsis thaliana wildtype-mutant experiment

Diana Trutschel[1,2], Stephan Schmidt[1], Ivo Grosse[2,3], Steffen Neumann[1]

June 25, 2015

[1]Leibniz Institute of Plant Biochemistry, Department of Stress and Developmental Biology,
Weinberg 3, 06120 Halle, Germany, sneumann@IPB-Halle.DE
[2]Martin-Luther-University Halle-Wittenberg, Institute of Computer Science,
Von-Seckendorff-Platz 1, 06120 Halle, Germany
[3] German Centre for Integrative Biodiversity Research (iDiv) Halle-Jena-Leipzig, Leipzig, Germany

Contents

1 Introduction **1**

2 Theory **2**

3 Tutorial **2**
3.1 Requirements 2
3.2 Wildtype-mutant experiment 3
3.3 Data 4
3.4 Results of the multivariate and univariate analysis 5

4 All R-functions provided in file 'multivariateDiffreport.R' **14**

1 Introduction

This document provides additional information for the article "How to exploit not-independent features in metabolomics for biomarker discovery". Here, we provide the R-code of the univariate and multivariate analysis of

Arabidopsis thaliana wildtype-mutant experiment for experimentalists. The xcms-Set of mass spectrometry data and the compound spectra analysed with the R-package CAMERA is given. Here, the multivariate analysis of related features of compound spectra is shown exemplary. Moreover, an advanced version of the xcms `diffreport()` function is provided for users. Additionally, we briefly explain all provided functions in the last section.

2 Theory

We propose an uncorrelated type of a multivariate test, suggested by (James (1954)), analysing compound spectra intensities in a multivariate manner in contrast to univariate analysis of single feature intensities. The extension of Student's t-test with unknown but equal variances becomes the Hotelling's T^2 in multiple dimensions. The univariate Welch (**?**) test allows different variances between the classes. The concept of a two-sample-test statistic on multidimensional data with unequal covariance matrices was introduced in (James (1954)). This test compares the difference of p-dimensional mean intensity vectors in relation to their common $p \times p$ covariance matrix. Observations of related features are then assumed to follow a multidimensional normal distribution.

To avoid the restriction of robust covariance matrix estimation, in the following only the diagonal entries are estimated. This simplification makes the covariance estimation more robust, and ignores the correlation in the test decision, which would make the approach prone to over fitting.

3 Tutorial

3.1 Requirements

The following packages are needed and have to be loaded, if necessary installed with `install.packages()` before:

- xcms
- CAMERA
- ellipse
- VennDiagram
- (multtest is implicit loaded within the process and has been installed from Bioconductor webpage)

```r
#load into library
library(xcms)
library(CAMERA)
library(VennDiagram)
library(ellipse)
```

Load the provided R-code from file the `multivariateDiffreport.R` with convenience functions under the GNU license.

```r
# This program is free software: you can redistribute it
# and/or modify it under the terms of the GNU
# General Public License as published by the Free
# Software Foundation, either version 3 of the License, or
# (at your option) any later version.
#
# This program is distributed in the hope that it will
# be useful, but WITHOUT ANY WARRANTY;
# without even the implied warranty of
# MERCHANTABILITY or FITNESS FOR A
# PARTICULAR PURPOSE.  See the
# GNU General Public License for more details.
#
# You should have received a copy of the
# GNU General Public License
# along with this program.  If not,
# see <http://www.gnu.org/licenses/>.


source('multivariateDiffreport.R')
ls()

## [1] "annotateDiffreportextend" "colVars"
## [3] "james_uncorr"             "uniAndmultiforceTests"
```

3.2 Wildtype-mutant experiment

The method is demonstrated on a dataset of a wildtype and a overexpression mutant line, available as MTBLS169[1]. The model plant *Arabidopsis thaliana*

[1] `http://www.ebi.ac.uk/metabolights/reviewerVzwz11PsCR`

Col-0 was used as plant material. For the genotype comparison Col-0 and a mutant were used, a transposon based activation tagged *A. th.* line from the TAMARA population (Schneider et al. (2005)). This particular mutant has an overexpression of the AT5G55880 - AT5G55890 genetic region with unknown function. Each population was grown under identical conditions.

The raw data files, the preprocessed peak matrix and the protocol descriptions have been submitted to the Metabolights repository (Haug et al. (2013)), and are available under the accession number MTBLS169 [2].

Using this tutorial only the file 'MTBLS169.Rdata' is needed. It includes the grouped `xcms-Set` ('xset') and the `xsAnnotate` ('an') object with identified features in feature groups by the package CAMERA (Kuhl et al. (2012)).

```
#load data
load(file='MTBLS169.Rdata')
ls()

## [1] "an"                          "annotateDiffreportextend"
## [3] "colVars"                     "james_uncorr"
## [5] "uniAndmultiforceTests"       "xset"
```

3.3 Data

The dataset comprised of seven *A. th.* Col-0 wildtype plants and seven plants of an *A. th.* overexpression line were analysed. In this case, the real effect is unknown, and here only a few exemplary results are described.

At first, we extract the feature intensities (logarithmised to remain normal distribution of intensities), the number of features and the experiment design out of the `xcms-Set`. Additionally, the `xsAnnotate`-object provides the number of compound spectra and their corresponding features.

```
#logarithmised feature intensities
#a matrix of number of features x number of measurments
vals<-log(groupval(xset, value="into",  "medret"), base=2)
#number of features
(Signals<-dim(vals)[1])

## [1] 2110
```

[2] http://www.ebi.ac.uk/metabolights/reviewerVzwz11PsCR

```r
#one-factorial experiment design
fac<-xset@phenoData[,"class"]
names(fac)<-colnames(vals)
fac

##  1  2  3  4  5  6  7  8  9 10 11 12 13 14
## MT MT MT MT MT MT MT WT WT WT WT WT WT WT
## Levels: MT WT

#number of annotated compound spectra
groupofpeaks<-an@pspectra
length(groupofpeaks)

## [1] 335
```

The data processing of the 14 samples results in a 2110 $\times$ 14 feature matrix, where CAMERA detected 335 compound spectra.

The separation of metabolites into a number of sub-molecules during ionization process may be exponentially distributed. at least groups with different sizes were annotated by CAMERA (Figure 1 left).

```r
#number of features in each compound spectra
howmany<-sapply(1:length(groupofpeaks),
                function(i){length(groupofpeaks[[i]])})
```

3.4 Results of the multivariate and univariate analysis

We use the proposed multivariate uncorrelated method to identify differential produced compound spectra. We evaluate univariate feature detection in comparison to multivariate compound spectra detection.

```r
#univariate tests for each feature
#multivariate tests for each compound spectra
pval<-uniAndmultiforceTests(vals,fac, groupofpeaks)
```

The function `uniAndmultiforceTests()` returns a list and a table. The list, here exemplary shown for the first list entry (first compound spectra), includes the results for each compound spectra. For each compound spectra the result of the multivariate method is returned separately from the results of the univariate method for each feature of the compound spectra. The

```r
plot(table(howmany),
     xlab="Compound spectra size",
     ylab="Number of compound spectra",
     cex.lab=1.5, cex.axis=1,
     #main="Distribution of compound spectra sizes"
     )
```

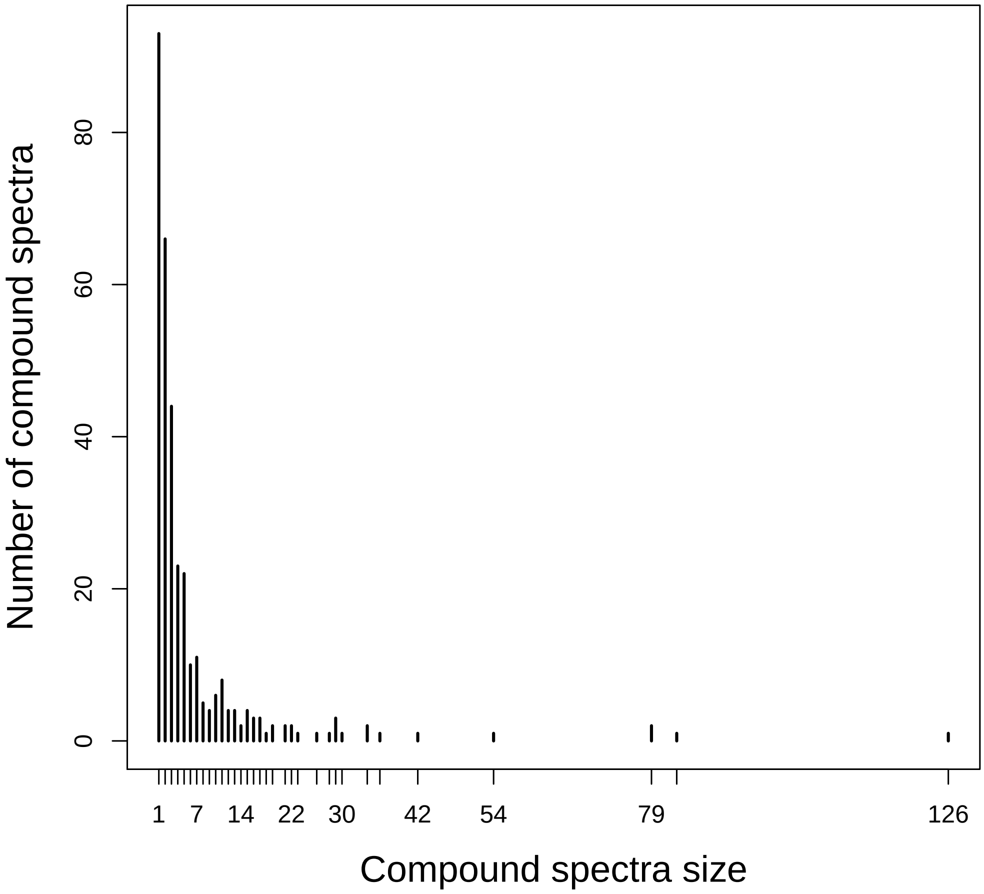

Figure 1. Distribution of compound spectra size using CAMERA annotation.

table, which lists the results for each feature, is exemplary shown in Table 1 for 10 features of two compound spectra.

```
#in a list format, p-values for each compound spectra
pval$l[[1]]

## $p.manov.force
## [1] 0.03844021
##
## $p.anov
##  [1] 0.01542879 0.04715554 0.41864939 0.28591391 0.05750393 0.69825364
##  [7] 0.03578760 0.03607826 0.03395289 0.03245133
```

	p.uni	p.multi	featnr	name	group.anno
1	0.02	0.04	437.00	237.1/1005	1
2	0.05	0.04	442.00	237.6/1005	1
3	0.42	0.04	479.00	244.1/1005	1
4	0.29	0.04	561.00	258.1/1005	1
5	0.06	0.04	653.00	274.1/1005	1
200	0.57	1.00	780.00	290.3/931	9
201	0.62	1.00	869.00	304.3/931	9
202	0.68	1.00	889.00	306.3/931	9
203	0.74	1.00	904.00	307.3/931	9
204	0.59	1.00	910.00	308.3/931	9

Table 1: Results of univariate and multivariate test listed for each feature

```
#for a given significance level
alph<-0.01
```

As shown in Figure 2 (left), at a significance level of $\alpha = 0.01$, 5 features are reported exclusively by the univariate method, while the multivariate approach detected 23 features exclusively. We present the number of detected features or compound groups, respectively, using a Venn Diagram to compare the results of both methods.

```
####################################################
###compare features                              ###
###which are detected with univariate and        ##
###which are detected with multivariate method   #
```

```r
################################################

#Significant features,
#which are detected with unvariate method
unisig<-which(pval.select[,"p.uni"]<=alph)
#number of univariate detected features
length(unisig)

## [1] 16

#Significant features,
#which are detected with multivariate method
multALLsig<-which(pval.select[,"p.multi"]<=alph)
#number of multivariate detected features
length(multALLsig)

## [1] 34

#identify the corresponding compound spectra number
wtmp<-which(!pval.select[unisig,"featnr"]%in%
              pval.select[multALLsig,"featnr"])
tmp<-pval.select[unisig,][wtmp,]
psnumber.uni<-unique(tmp[,"group.anno"])


#venn diagram compares the features,
#which are detected with both methods
venn.diagram(
  list("univariate"=pval.select[unisig,"featnr"],
      "DiagJames"=pval.select[multALLsig,"featnr"]),
       col=c("black","blue"),sub.col=c("black","blue"),
      filename="test_7vs7_real.png", imagetype="png",
      main="Compare evaluate differential signals
      with uni and uncorrelated multivariate method",
      cex=4,cat.cex=2.5,
      cat.dist=c(0.03, 0.03) , cat.pos=c(-25,170)
)

## [1] 1
```

At the compound spectra level it is shown in Figure 2 (right), that 3

compound spectra are exclusively found by the multivariate approach, and 4 compound spectra (with the pseudo spectrum number 11, 17, 55, 237) are found with at least one feature, which is exclusively detected by the univariate approach.

```
#####################################################
###compare features groups                       ####
###which are detected with univariate and        ###
### and with later group assignment              ##
###which are detected with multivariate method  #
################################################

#Significant features groups,
#which are detected with unvariate method
#and later group assignment
postAnnoDiffs<-unique(pval.select[unisig,"group.anno"])
length(postAnnoDiffs)

## [1] 10

#Significant features groups,
#which are detected with multivariate method
Annodiffs<-unique(pval.select[multALLsig,"group.anno"])
length(Annodiffs)

## [1] 9

#identify the corresponding compound spectrum number
onlymulti<-which(pval.select[,"group.anno"]%in%
             Annodiffs[which(!Annodiffs%in%postAnnoDiffs)])
psnumber.multi<-unique(pval.select[onlymulti,"group.anno"])


#venn diagram compare the features groups,
#which are detected with both methods
venn.diagram(
  list("univariate.min"=postAnnoDiffs,
      "DiagJames"=Annodiffs),
      col=c("black","blue"),sub.col=c("black","blue"),
      filename="testt_group_7vs7_real.png", imagetype="png",
      main="Compare evaluate differential groups
```

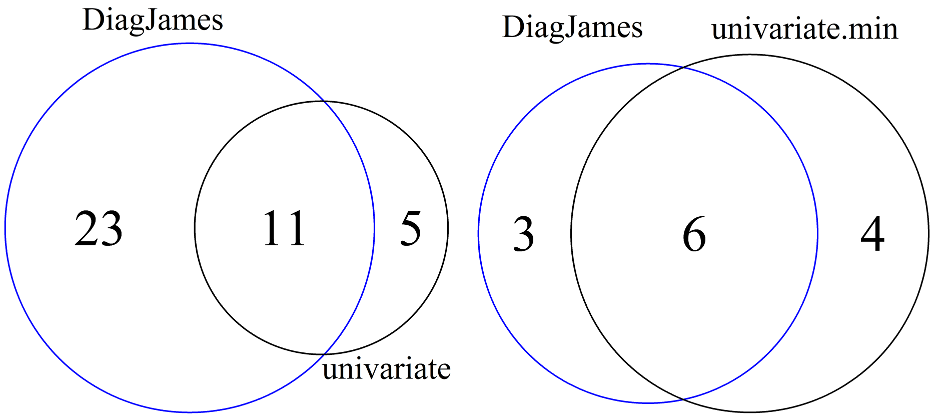

Figure 2. Venn Diagram of differential features and compound spectra in the wildtype-mutant experiment for the significance level of $\alpha = 0.05$. Left: number of *features* detected by univariate and multivariate method. Right: number of *compound spectra* detected by the multivariate method, compared to the number of compound spectra where at least one feature was detected univariately.

```
       with uni and multivariate uncorrelated method",
       cex=4,cat.cex=2.5,
       cat.dist=c(0.04, 0.06) , cat.pos=c(160,-165)
     , inverted=TRUE
)
```

All 3 compound spectra (with pseudo spectrum number 61, 93, 194) are only found by multivariate method are small groups with only two or rather 3 features. Figure 3 shows the pseudo spectra for each of the compound spectra. The pseudo spectra of 4 compound spectra detected by univariate method are shown in Figure 4. Only one of the compound spectra is a small group with only 2 features, the others have a size of 15, 17 and 35. Here, it has been shown, that compound spectra, which are exclusively found by the multivariate method, have only little features, where compound spectra with features, which are exclusively found by the univariate method, are often groups with many features. These results underline the advantage of detecting differential metabolites with the multivariate approach for noisy data.

Additionally, we provide an extension of the common used `diffreport()` of the CAMERA package. This function combines the `diffreport()` of an

```r
par(lwd=1, cex.lab=2, cex.axis=1.5)
par(cex=7, oma=c(0,0,0,0), mar=c(4,5,1,1))
par(mfrow=c(3,1))
plotPsSpectrum(an, psnumber.multi[1],
               title="", log=TRUE, mzrange=c(0,250),
               lwd=3, maxlabel=5,  ylab="log Intensity")
plotPsSpectrum(an, psnumber.multi[2],
               title="", log=TRUE, mzrange=c(0,300),
               lwd=3, maxlabel=5,  ylab="log Intensity")
plotPsSpectrum(an, psnumber.multi[3],
               title="", log=TRUE, mzrange=c(0,250),
               lwd=3, maxlabel=5,  ylab="log Intensity")
```

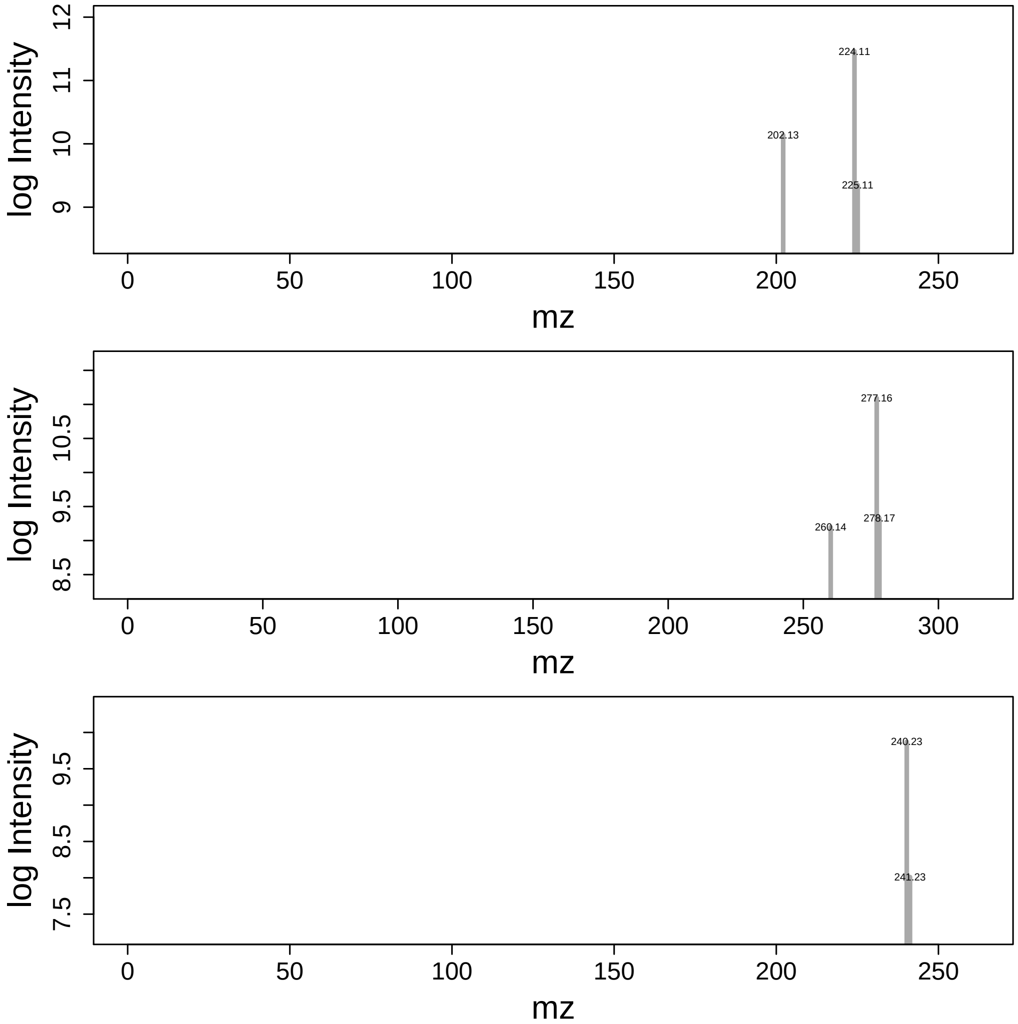

Figure 3. Three compound spectra detected exclusively by the multivariate approach. The retention times are 328s, 180s and 506s, respectively.

```
par(lwd=1, cex.lab=2, cex.axis=1.5)
par(cex=7, oma=c(0,0,0,0), mar=c(4,5,1,1))
par(mfrow=c(4,1))
plotPsSpectrum(an, psnumber.uni[1],
               title="", log=TRUE, mzrange=c(0,500),
               lwd=3, maxlabel=50,  ylab="log Intensity")
plotPsSpectrum(an, psnumber.uni[2],
               title="", log=TRUE, mzrange=c(0,750),
               lwd=3, maxlabel=50,  ylab="log Intensity")
plotPsSpectrum(an, psnumber.uni[3],
               title="", log=TRUE, mzrange=c(0,620),
               lwd=3, maxlabel=50,  ylab="log Intensity")
plotPsSpectrum(an, psnumber.uni[4],
               title="", log=TRUE, mzrange=c(0,250),
               lwd=3, maxlabel=5,  ylab="log Intensity")
```

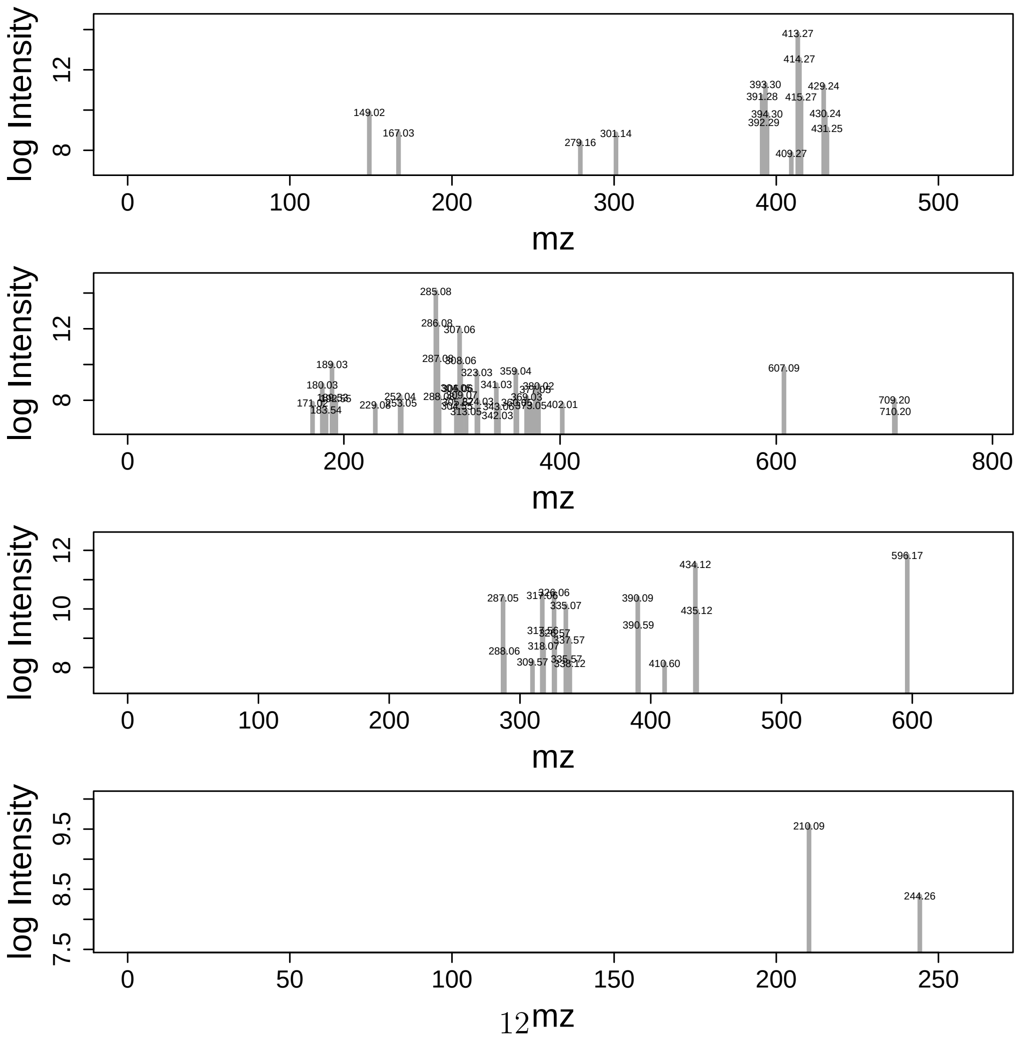

Figure 4. Four compound spectra contain features, which are detected exclusively by the univariate approach. The retention times are 233s, 533s, 564s and 1023s, respectively.

XCMS-set with the results of the multivariate analysis. We show in Table 2 - Table 6 the extended diffreport for all features of the compound spectra, which are exclusively detected by the multivariate method.

```
annodiff <- diffreport(xset, sortpval=FALSE,
                       class1 = levels(sampclass(xset))[1],
                       class2 = levels(sampclass(xset))[2])
diffextend<-annotateDiffreportextend(annodiff,pval$t)
```

	name	fold	tstat	pvalue	mzmed	mzmin	mzmax
202.1/328	M202T328	1.72	1.86	0.09	202.13	202.12	202.13
224.1/328	M224T328	1.59	1.88	0.09	224.11	224.10	224.11
225.1/328	M225T328	1.57	1.72	0.12	225.11	225.10	225.11
240.2/507	M240T507	1.36	-2.47	0.03	240.23	240.23	240.23
241.2/507	M241T507	1.50	-2.78	0.02	241.23	241.23	241.23
260.1/181	M260T181	1.82	2.14	0.07	260.14	260.13	260.14
277.2/181	M277T181	1.86	2.21	0.06	277.16	277.16	277.17
278.2/181	M278T181	1.86	2.22	0.06	278.17	278.16	278.17

Table 2: Extended Diffreport (column 1-7) of the wildtype-mutant-experiment results

	rtmed	rtmin	rtmax	npeaks	dummy
202.1/328	328.13	327.31	330.63	14.00	14.00
224.1/328	328.01	326.97	330.30	14.00	14.00
225.1/328	328.01	327.31	330.30	14.00	14.00
240.2/507	506.73	506.14	509.18	14.00	14.00
241.2/507	506.87	506.14	509.18	14.00	14.00
260.1/181	180.58	179.41	183.42	14.00	14.00
277.2/181	180.90	179.71	183.75	14.00	14.00
278.2/181	180.75	179.07	183.75	14.00	14.00

Table 3: Extended Diffreport (column 8-12) of the wildtype-mutant-experiment results

	1	2	3	4	5	6
202.1/328	25270.58	23135.53	81017.85	15500.08	15731.32	21228.74
224.1/328	94984.24	84169.45	256686.83	54999.96	66468.50	70782.76
225.1/328	11480.20	9681.72	32263.19	6580.19	8268.55	8255.06
240.2/507	19383.66	24124.46	34571.04	23260.09	21036.83	28649.24
241.2/507	3013.39	3843.36	5744.47	3665.02	3607.26	4239.30
260.1/181	9866.12	16205.60	10817.91	17179.43	17324.90	9968.56
277.2/181	65625.21	117431.08	74120.65	120513.85	115215.61	69386.17
278.2/181	11267.09	18155.43	12351.20	20019.61	20664.03	11448.24

Table 4: Extended Diffreport (column 13-18) of the wildtype-mutant-experiment results

	7	8	9	10	11
202.1/328	17995.72	30112.44	49895.47	52753.02	46680.06
224.1/328	62061.44	114329.85	173992.68	173491.88	158852.38
225.1/328	7522.82	13790.78	18627.57	19881.31	19715.37
240.2/507	26123.71	14498.58	23767.95	15065.94	18030.10
241.2/507	4335.89	1684.04	3559.05	2013.71	2630.38
260.1/181	10221.09	39010.75	14409.40	13381.65	15529.68
277.2/181	71280.11	260810.99	106459.46	95590.36	102954.62
278.2/181	11917.06	46581.99	17456.63	15711.18	17323.90

Table 5: Extended Diffreport (column 19-23) of the wildtype-mutant-experiment results

4 All R-functions provided in file 'multivariateDiffreport.R'

included several R functions, listed here:

1. `colVars()`

   provided by David Brahm (brahm@alum.mit.edu)

   Input: a matrix x

   Output: variances of each column of the matrix x

2. `james_uncorr()`

   multivariate method proposed by (James (1954))

	12	13	14	group.anno	p.multi
202.1/328	26269.22	59754.75	78915.12	61	0.01
224.1/328	86536.67	196135.13	196784.57	61	0.01
225.1/328	10418.97	24344.26	24803.56	61	0.01
240.2/507	27289.96	13599.37	17685.66	194	0.00
241.2/507	4261.28	1827.89	2985.90	194	0.00
260.1/181	21202.53	18248.35	44739.98	93	0.01
277.2/181	156814.67	129664.71	325034.79	93	0.01
278.2/181	24554.02	23222.19	52110.68	93	0.01

Table 6: Extended Diffreport (column 24-28) of the wildtype-mutant-experiment results

function for orignal test is provided by (Tsagris [3])

we adapt to an uncorrelated type of the test

Input: matrix of feature intensities, one for sample class 1 and one for sample class 2, significance level α

Output: p-value of significance for multivariate uncorrelated test of mean vector comparison of the two sample classes

3. `uniAndmultiforceTests()`

   Input: matrix of feature intensities (features x samples), factorial design, compound spectra

   Output: list of results: (#multivariate analysed groups, p values listed for each group, p values in a matrix)

4. `annotateDiffreportextend()`

   Input: results of function `differport()`, multivariate analysis results of function `uniAndmultiforceTests()`

   Output: matrix of combined results

References

Kenneth Haug, Reza M Salek, Pablo Conesa, Janna Hastings, Paula de Matos, Mark Rijnbeek, Tejasvi Mahendraker, Mark Williams, Steffen

[3] `https://www.academia.edu/1887808/Multivariate_statistical_functions_in_R`

Neumann, Philippe Rocca-Serra, Eamonn Maguire, Alejandra Gonzlez-Beltrn, Susanna-Assunta Sansone, Julian L Griffin, and Christoph Steinbeck. MetaboLights–an open-access general-purpose repository for metabolomics studies and associated meta-data. *Nucleic Acids Res*, 41 (Database issue):D781–D786, Jan 2013. doi: 10.1093/nar/gks1004. URL http://dx.doi.org/10.1093/nar/gks1004.

G.S. James. Tests of linear hypotheses in univariate and multivariate analysis when the ratios of the population variances are unknown. *Biometrika*, (41(1/2)):19–43, 1954.

C. Kuhl, R. Tautenhahn, C. Bttcher, R. Larson, and S. Neumann. CAMERA: An integrated strategy for compound spectra extraction and annotation of LC/MS data sets. *Anal Chem.*, 84 (1):283–289, 2012. doi: 10.1021/ac202450g. URL http://pubs.acs.org/doi/abs/10.1021/ac202450g.

Anja Schneider, Thomas Kirch, Tamara Gigolashvili, Hans-Peter Mock, Uwe Sonnewald, Rüdiger Simon, Ulf-Ingo Flügge, and Wolfgang Werr. A transposon-based activation-tagging population in Arabidopsis thaliana (TAMARA) and its application in the identification of dominant developmental and metabolic mutations. *FEBS Lett*, 579(21):4622–4628, Aug 2005. doi: 10.1016/j.febslet.2005.07.030. URL http://dx.doi.org/10.1016/j.febslet.2005.07.030.

Michail T. Tsagris. *Multivariate statistical functions in R*. URL https://www.academia.edu/1887808/Multivariate_statistical_functions_in_R.
